# Dosage imbalance of B- and C-class genes causes petaloid-stamen relating to F_1_ hybrid variation

**DOI:** 10.1186/s12870-018-1562-4

**Published:** 2018-12-07

**Authors:** Jing Liu, Chao-Qun Li, Yang Dong, Xia Yang, Yin-Zheng Wang

**Affiliations:** 1 State Key Laboratory of Systematic and Evolutionary Botany, Institute of Botany, Chinese Academy of Sciences, 20 Nanxincun, Xiangshan, Beijing, 100093 China; 2 University of Chinese Academy of Sciences, Beijing, 100049 China

**Keywords:** ASE analyses, B-class genes, Dosage imbalance, Floral symmetry, Organ identity, Parallel pathways, Petaloid-stamen, *Petrocosmea*

## Abstract

**Background:**

Great advances have been achieved in our understanding of flower development and evolution since the establishment of the ABC model. However, it remains a challenge to define the exact context of organ identity in the component interactions of the ABC model.

**Results:**

Through hybridization, we detected a homeotic mutant in *Petrocosmea* (Gesneriaceae) uniquely displayed by the ‘petaloid-stamen’ in the third whorl with petal identity. Comparative Real-time PCR analyses demonstrate that both two B-class genes *DEF2* and *GLO* are excessively expressed while the transcripts of the C-class gene *PLE* are reduced in the third floral whorl in the mutant compared to that in the wild-type F_1_ hybrids. Further allele-specific expression (ASE) analyses indicate that an allele-specific change in *PgPLE* might be responsible for up-regulation of both B-class genes and down-regulation of the C-class gene in the petaloid-stamen mutants.

**Conclusions:**

Our findings suggest that the petaloid-stamen is consequent upon an evident dosage imbalance between B- and C-class products that is probably triggered by a *cis*-regulatory change. In addition, the genetic pathway for the floral organ identity might be in parallel with that for the floral symmetry. The extreme variation in hybrids further suggests that interspecific hybridization may represent a major factor for evolutionary innovation and diversification in plants.

**Electronic supplementary material:**

The online version of this article (10.1186/s12870-018-1562-4) contains supplementary material, which is available to authorized users.

## Background

Flowers, as a key innovation in angiosperm, have been the focus of mankind owing to their evolutionary success, unique beautiful appearance and commercial values. About two decades ago, the canonical flower development model, known as the ABC model, was proposed based on homeotic mutants which refer to the transformation of the organ identity from one to another at particular whorl produced in *Arabidopsis thaliana* and *Antirrhinum majus* [1]. The organ identity genes corresponding to the three classes of mutant phenotypes A, B and C were gradually cloned based on homeotic mutants in *A. thaliana* and *A. majus* [2–9]. In the ABC model, the A-class gene alone determines the sepal identity in the first whorl; A- together with B-class genes specify the petal identity in the second whorl; B- combined with C-class genes define the stamen in the third whorl; and the C-class gene itself controls the carpel development and is mutually antagonistic to A-class genes [1]. Afterward, newly cloned genes respectively classified as D-class, specifically controlling ovule development, and E-class, contributing to flower organ identity together with other classes, were added to the ABC model, thus, yielding the ABCDE model [10, 11]. At the same time, a quartet model was proposed based on the interaction among different class genes [12]. However, progresses in universal gene expression and phenotypic analyses of A-class mutants in diverse species make the A function blur in controlling petal identity. Difficulty in defining A-class genes working as the A function compared to the conserved B and C function in flower development promotes the emergence of (A)BC model [13].

Although great advances have been achieved in our understanding of flower development, there are still many questions unanswered from these themes because of enormous diversity of angiosperm flowers. Sometimes, different genetic pathways may underlie similar floral phenotypes, while similar developmental programs could produce diverse floral forms. For example, petalody of the first whorl, i.e. tepal, is associated with the expansion of B-class transcripts in *Lilium* [14], but the petalody of the first whorl in *Aquilegia* is not related to B-class genes at all [15, 16]. The petalody or petaloid-stamen of the third floral whorl is more complicated than the situation of the first whorl because it is potentially related to both B- and C-class gene activities. In fact, the earliest descriptions of floral mutants are the petaloid-stamen mutant in which petals replace stamens in the third whorl, also named double flowers, going back to ancient Greece, Rome and China more than two thousand years ago [17]. In China, the peonies with petaloid-stamens, i.e. double flower peonies, were known and selected in 750 AD [18]. From then on, the petaloid-stamen flower has been widely found in a large number of groups, including *Daucus carota* (Apiaceae), *Gossypium* (Malvaceae), *Nicotiana tabacum* (Solanaceae), *Caltha palustris* (Ranunculaceae), *Plantago* (Plantaginaceae), *Brassica juncea* (Brassicaceae), *Petunia* (Solanaceae), *Vinca minor fl. Pl.* (Apocynaceae), *Canna indica* (Cannaceae), *lilium* (Liliaceae), *Camellia japonica* (Theaceae), *Rosa hybrid* (Rosaceae) and *Torenia fourmieri* (Scrophulariaceae) [19–33]. Homeotic mutants in *A. thaliana* and *A. majus* play an important role in the intersection of genetic and development as the basis for the establishment of the classical ABC model. However, it is far from enough to fully understand the diversification of floral morphology, as well as the naturally frequently occurred petaloid-stamen mutant, in angiosperms. Genetic and molecular studies of the petaloid-stamen mutant in non-model organisms would provide a new insight into the genetic and evolutionary origin of particular floral organ types or novel mechanism underlying specific phenomena that is not present in current model systems.

*Petrocosmea* belongs to Gesneriaceae, a member of Asteridae in core eudicots, and consists of 47 species with uniform chromosome number of 2n = 34. They are mostly distributed in southwestern China. The zygomorphic flowers of *Petrocosmea* exhibit a wide range of morphological variation mainly in the upper lip, i.e. the two dorsal petals [34]. During the artificial hybridization process, we find several mutant plants with petaloid sterile stamens in the third whorl of the homoploid F_1_ hybrid *P*. *glabristoma* × *P. sericea* flowers in glasshouse. The normal F_1_ hybrids have a phenotype falling somewhere between the two parents. It is interesting that the petaloid-stamen flowers with changed floral organ identity in the third whorl have not any obvious alteration in other organ identity as well as in dorsal-ventral asymmetry, i.e. zygomorphy, in both the second and third floral whorls. Therefore, this petaloid-stamen mutant presents an ideal candidate to clarify the molecular basis of flower organ identity and the genetic relationship between floral organ identity and symmetry in Asteridae of core eudicots.

In this study, we selected the *Petrocosmea* hybrid as a model to perform our researches with multiple experimental approaches. Our results show that the petaloid-stamen is induced by altered homeotic gene expression in F_1_ hybrids, i.e. up-regulation of B-class and down-regulation of C-class genes. We further find that the C-class alleles are differentiated from the wild-type to mutant F_1_ hybrid flowers, which may trigger the change of both C-class and B-class gene expression. Our findings suggest that the petaloid-stamen mutant is associated with an evident dosage imbalance between B- and C-class gene products that might be caused by an allele-specific regulatory change, without affecting dorsal-ventral asymmetry. In addition, our hybridization experiments in related species of *Petrocosmea* suggest that interspecific hybridization likely generates extreme deviants which as ‘hopeful monsters’ would be a great source of evolutionary innovation. Our results shed new light on our understanding of the interaction of B- and C-class genes, the genetic relationship between floral organ identity and symmetry genes as well as the role of hybridization in plant evolution.

## Methods

### Plant materials and crossing experiment

*P. glabristoma* (23 individual plants) and *P. sericea* (27 individual plants) were used in this study as the female and male parent, respectively, to produce the interspecific F_1_ hybrid *P. glabristoma* × *P. sericea*. About 184 F_1_ hybrid plants were developed, in which 19 plants exhibit severe petaloid-stamen phenotype uniformly, the rest 165 plants produce wild-type flowers. Both parents and other related *Petrocosmea* species were grown in the glasshouse of Institute of Botany, Chinese Academy of Sciences (IBCAS), Beijing, China, which provides a moist and shady micro environment. The photoperiod was controlled under the natural photoperiod in Beijing with a day/night temperature regime of 25 °C/20 °C, respectively. The crossing procedure was as follows: fresh pollen was collected as a pool from newly blooming flowers of the male individuals and transferred to the stigma of emasculated female flowers. The remaining pollens were stored at 4 °C and used to fertilize the same stigmas repeatedly about 1-2 times per day during the next 4-5 days. After about two months, the mature seeds were collected.

The harvested F_1_ hybrid seeds were surface-sterilized in 70% (*v*/v) ethanol for 3 min, washed with sterilized water once, soaked in 2.5% sodium hypochlorite solution for 3 min, and finally washed thoroughly with sterilized water for three times. The sterilized seeds were germinated on Murashige and Skoog (MS) medium supplemented with 3% (*w*/*v*) sucrose and 0.8% (w/v) agar at 25 °C under a 12 h-light and 12 h-dark cycle conditions. The seedlings with 2-3 true leaves were transplanted to a 10 cm pot containing the mixture of vermiculite, perlite and pindstrup substrate (Pindstrup, Denmark) (1:1:1), and grown in glasshouse under the same condition as the parents. The genuine F_1_ hybrid offspring were confirmed by detailed morphological characterization followed by single nucleotide polymorphism (SNP) genotyping on the heterozygous sites of *CYC-*like locus.

### DNA extraction and *CYC*-like gene isolation

Total genomic DNAs of both parents and their F_1_ hybrids were extracted from fresh young leaves with a DNeasy Plant Mini Kit (TIANGEN, Beijing, China) following the manufacturer’s instruction. The *CYC*-like genes were amplified from the genomic DNA using ortholog-specific primers designed based on *CYC*-like sequences cloned in *Petrocosmea* (Additional file 1: Table S1) [35]. The PCR products were sequenced directly.

### RNA extraction and A-, B-, C-class gene isolation

Total RNAs were extracted from young inflorescences and different floral organs of *P*. *glabristoma*, *P. sericea* and their F_1_ hybrids using a SV Total RNA Isolation System (Promega, Madison, WI, USA) according to the manufacturer’s instruction. The first-strand cDNAs were synthesized using a RevertAid H Minus First-Strand cDNA Synthesis Kit (Thermo Scientific, Rochester, NY, USA) according to the manufacturer’s instructions. The A-, B- and C-class floral organ identity genes were isolated from young inflorescences cDNA by reverse transcription PCR (RT-PCR) using gene-specific primers (Additional file 1: Table S1). These primers were designed according to conserved sequences from *N. tabacum*, *A. thaliana*, *A. majus* and *Petunia hybrida*. The PCR product of each gene from parental species was first sequenced directly. When double peaks appeared at certain positions, the PCR products were cloned into the pEasy-T1 simple vector (TransGen Biotech, Beijing, China) and sequenced. Sequences isolated in this article can be found in the Additional file 2.

### Phylogenetic analyses

To confirm homologues, we first carried out BLAST in the National Center for Biotechnology Information (NCBI) database using the A-, B- and C-class gene sequences isolated from the two parents as queries, respectively. Sequences isolated in this study belonging to MADS-box and AP2/EREBP family, respectively, were added to separate datasets of sequences. Sequences representing major subfamily members of A-, B- and C-class MADS-box and AP2/EREBP family genes from *A. thaliana* and *A. majus* were retrieved from NCBI database. The nucleotide sequences were converted into amino acid sequences using the standard genetic code for translation and aligned with manual adjustments using Mega 6.0 software [36]. Further, Neighbor-joining (NJ) analyses were carried out using Mega 6.0 software [36]. Bootstrap values calculated for 1000 replicates were conducted to access the statistical reliability of the inferred topology using Mega 6.0 software [36]. Similarly, phylogenetic analyses of *CYC*-like genes were conducted with a sequence matrix including *CYC*-like TCP sequences from *Petrocosmea* using Mega 6.0 software [36].

Sequence data used in this article can be found in the GenBank data library under following accession numbers: AmSQU, X63701; AtAP1, Z16421; AmDEF, X52023; AtAP3, D21125; AmGLO, AB516403; AtPI, AF115825; AmPLE, AB516404; AtAG, NM_118013; AtAGL67, NM_001334807; AtAGL66, NM_106447; AtTOE3, NM_126118; AtTOE1, NM_001202696; AtTOE2, NM_001203647; AtSNZ, NM_179982; AtSMZ, NM_001084821; AtAIN, NM_119937; AmLIP1, AY223518; AmLIP2, AY223519; AtAP2, NM_001204009; PsiCYC1C, KT596755; PsiCYC1D, KT596756; PsiCYC2A, KT596757; PsiCYC2B, KT596758; PgCYC1C, KT596755; PgCYC1D, KT596756; PgCYC2A, KT596757; PgCYC2B, KT596758; *Leucocarpus perfoliatus* DEFA, AY530540.1; *Leucocarpus perfoliatus* DEFB, AY530544.1; *Mimulus kelloggii* DEFA, AY530541.1; *Mimulus kelloggii* DEFB, AY530545.1; *Lantana camara* DEF, HQ853380.1; *Verbena officinalis* DEF, AY524009.1; *Lophospermum atrosanguineum* DEF, JQ173625.1; *Streptocarpus hybridus* DEF, HQ853387.1.

### Real-time PCR expression analyses

For sampling, both the parental and F_1_ hybrid flowers were pooled from different individual plants, respectively. As for the mutant F_1_ hybrids, they show uniform characters in mutation, i.e. petalody in the third floral whorl. The flower used for A-, B- and C-class gene expression analyses, just before anthesis, were divided into six parts: sepals, dorsal petals, lateral petals, ventral petal, (petaloid) stamens (the third floral whorls were sampled as a whole, due to the extremely tiny vestigial dorsal and lateral staminodes) and carpel, at low temperature. It should be noted that the third floral whorl, used for *CYC*-like gene expression analyses, was divided into dorsal, lateral and ventral organs, due to the special function of *CYC*-like genes in repressing dorsal organ development. But, the division of the third floral whorl organs is not suitable for A-, B- and C-class genes’ expression analyses because the remarkable difference of the dorsal, lateral and ventral organs in size would severely affect the accuracy and precision of the expression level analyses among the three parts. The collected samples were immediately frozen in liquid nitrogen, and stored at − 70 °C until use. RNA extraction and cDNA synthesis were conducted as described in the ‘RNA extraction and A-, B-, C-class gene isolation’ section. Real-time PCR was carried out using SYBR Premix ExTaq (TaKaRa, Dalian, China) with an Applied Biosystems StepOne Plus Real-time PCR System (AB Applied Biosystems, Beijing, China). Gene-specific primers were used to amplify respective genes from each sample (Additional file 1: Table S2). It is noteworthy that in Real-time PCR, two alleles for each gene in F_1_ hybrids were indiscriminately amplified using a pair of primers that designed in the conserved regions. The specificity of all primers was confirmed by directly sequencing PCR products. For allele-specific expression (ASE) analyses, primers were designed surrounding a fixed SNP site in the F_1_ hybrids and promising the most 3′ end nucleotide of one of the paired primers were divergent between alleles (Additional file 1: Table S3). Allele specific amplification of each pair of primers was tested by directly sequencing PCR products. The amplification efficiency of all primers used for Real-time PCR and ASE analyses was confirmed by standard curve according to 3-7 dilute series. The primers were used for expression analyses when their amplification efficiency was within the range of 95-105%. The Real-time PCR reaction conditions were as follows: initial denaturation for 30s at 95 °C; 40 cycles amplification of 10 s at 95 °C and 40 s at 60 °C, and a final melting curve cycle of 30s at 95 °C, 30s at 60 °C, and 30s at 95 °C. *ACTIN* gene was amplified as an internal control under the same conditions as target genes. StepOne Software v2.3 was used to collect data and carry out the statistical analyses (AB Applied Biosystems, Beijing, China). The relative expression level was determined by a Delta CT method to normalize the expression level of target gene to that of *ACTIN* of the same organs directly [37]. The data shown were the average of three biological replicates with each including three technical replicates. The expression difference significance among the two parents and wild-type versus mutant F_1_ hybrids were tested using the LSD test (*P* < 0.05) of SPSS 16.0 software (SPSS Inc.). When the mean values are significantly different between the mutants and their parents as well as their wild-type counterparts, asterisks were marked above corresponding column.

### Scanning electron microscopy

Mature flowers were fixed in FAA solution containing 50% (*v*/v) ethanol, 5% (v/v) formaldehyde and 10% acetic acid (v/v) at 4 °C overnight. The flowers were transferred into 70% ethanol and dissected into different organs. Then, the floral organs were dehydrated through a gradient ethanol (70, 80, 90, 95 and 100%) and transferred to a gradient isoamyl acetate/ethanol (1:3, 1:1 and 3:1 v/v). At last, the samples were transferred to 100% isoamyl acetate, dried using CO_2_ critical-point method and subjected to scanning electron microscopy (SEM, S-4800, FESEM).

## Results

### Phenotypic characterization of the parents and F_1_ hybrid offspring

**Fig. 1:**
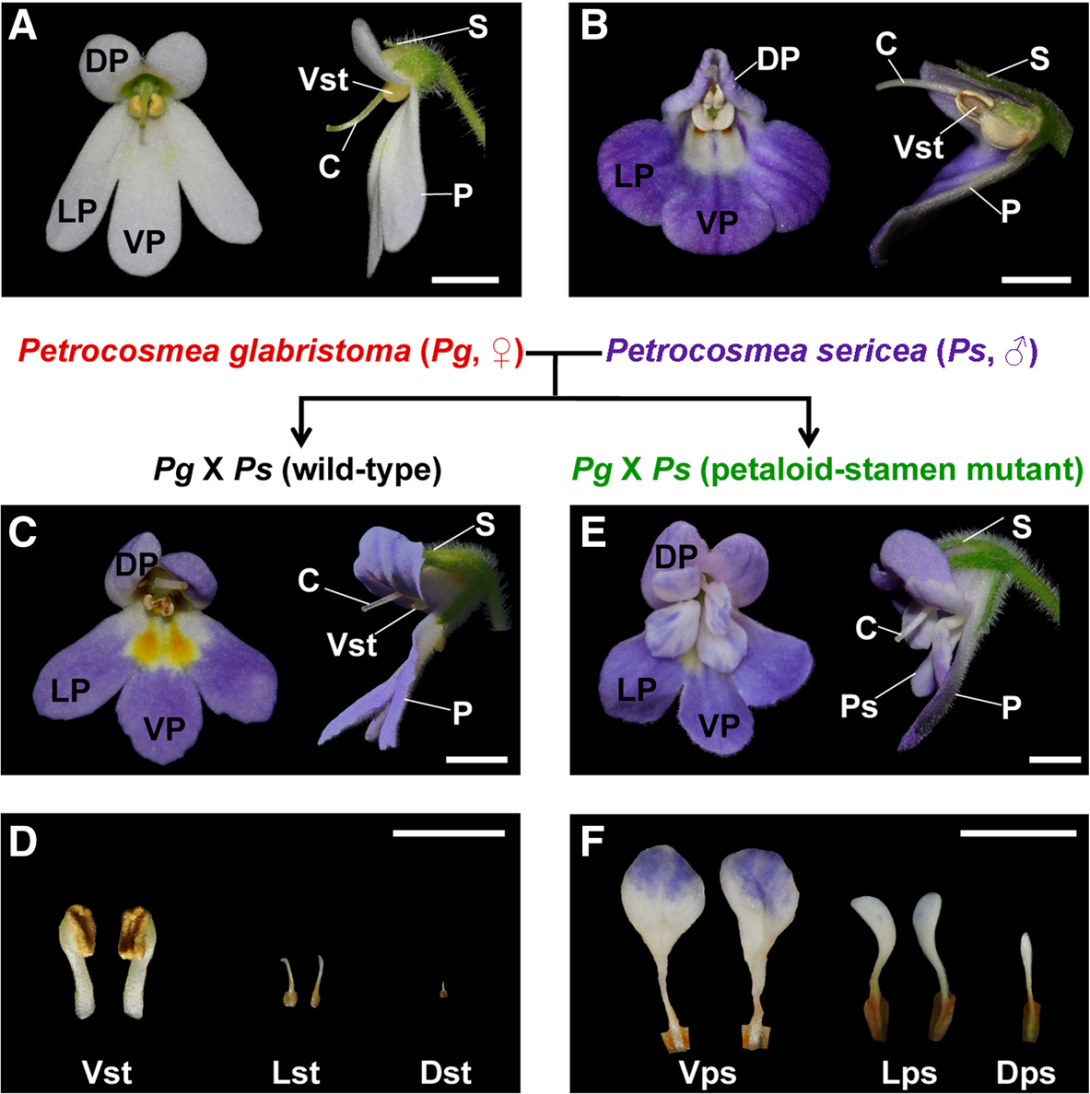
Flower morphology of *Petrocosmea glabristoma* (♀), *P. sericea* (♂) and their F_1_ hybrids. Both *P*. *glabristoma* (**a**) and *P. sericea* (**b**) produce zygomorphic and whorl-ranged perfect flowers which are mainly different from each other in the shape and size of dorsal petals in the second whorl and the shape of filaments in the third whorl. The F_1_ hybrid flowers are generally intermediate between the two parents in morphology. Some flowers of the F_1_ hybrids have normal stamens biased to the maternal parent (**c**), while others have stamens replaced by petaloid organs (**e**). **d** Dissected third floral whorl organs of the wild-type F_1_ hybrid flower with two fertile ventral stamens, two aborted lateral staminodes and one vestigial dorsal staminode. The stamens or staminodes increase in size from the dorsal to the ventral. **f** Dissected third floral whorl organs with five petaloid-stamens of the mutant F_1_ hybrid flower. These petaloid-stamens are different in size similar to the stamens and staminodes in corresponding position of the wild-type flower. For each flower, both the front (left) and side view (right) are shown. Dp, dorsal petal; Lp, lateral petal; Vp, ventral petal; S, sepal; P, petal; Vst, ventral stamen; C, carpel; Ps, petaloid stamen. Lst, lateral staminode; Dst, dorsal staminode; Vps, ventral petaloid-stamen; Lps, lateral petaloid-stamen; Dps, dorsal petaloid-stamen. Bar, 0.5 cm

The parental species *P*. *glabristoma* and *P. sericea* are similar in vegetative trait with rosette habit (Additional file 1: Figure S1A, B). In addition, they both have zygomorphic flowers with two smaller dorsal petals (upper lips) versus one ventral and two lateral larger petals (lower lips) in the second whorl and one dorsal and two lateral staminodes versus two fertile ventral stamens in the third whorl (Fig. 1a, b). However, the upper lip is extended upward and reflected backward and about half of the lower ones in size in *P*. *glabristoma*, but extended forward and extremely reduced in size, and specialized into a carinate-plicate structure in *P. sericea* (Fig. 1a, b). The filaments of the fertile ventral stamens are straight and linear in *P*. *glabristoma*, but geniculate with the middle part swollen in *P. sericea* (Fig. 1a, b).

The F_1_ hybrids are very similar to their parents in vegetative traits (Additional file 1: Figure S1). In floral trait, the F_1_ hybrids are biased to *P*. *glabristoma* in the upper lip reflected backward and the ventral filaments straight and linear but inclined to *P. sericea* in corolla color and shape of the ventral petal (Fig. 1a, c, d). In the naturally occurred mutant F_1_ hybrids, all stamens and staminodes are transformed to petal-like organs, i.e. petaloid-stamens, while other floral organs are similar to those of wild-type F_1_ hybrids (Fig. 1c, e, f). The severe petaloid-stamen mutants are uniform with only slight difference in size corresponding to flower size. In addition, the petal-like organs corresponding to the dorsally and laterally aborted and ventrally fertile stamens of wild-type F_1_ hybrids increase in size, indicating that the floral symmetry of the mutant is unchanged in the third whorl (Fig. 1d, f).

### Cell morphology in different floral organs of the wild-type and petaloid-stamen mutant flowers

**Fig. 2:**
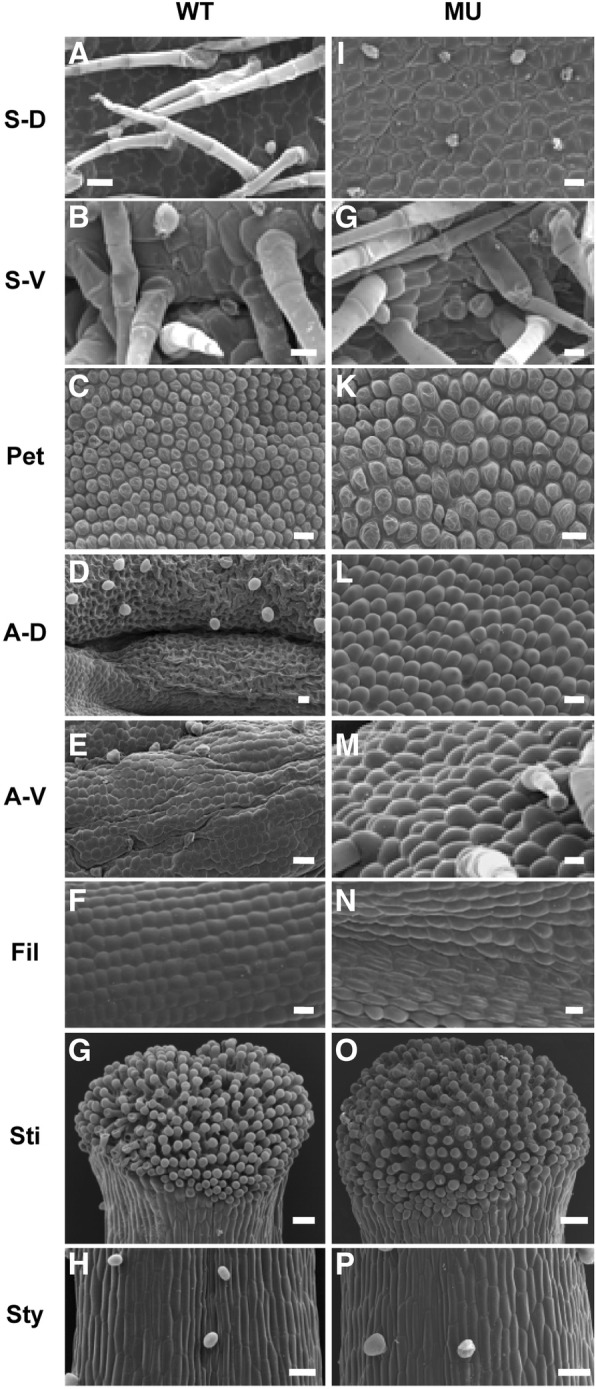
Epidermal cell morphology of *Petrocosmea glabristoma* × *Petrocosmea sericea* F_1_ hybrid flowers. **a**-**h** Epidermal cells of different floral organs of wild-type flowers. **i**-**p** Epidermal cells of different floral organs of mutant flowers. The sepal of both wild-type and mutant flowers is comprised of flat cells in both the dorsal (**a**, **i**) and ventral epidermis (**b**, **j**). The petal epidermis of both types of flowers is composed of conical cells (**c**, **k**). In the wild-type flower, epidermal cells in the dorsal side of anthers (**d**) are reticular, while the filament (**e**) and the ventral side of anthers (**f**) are full of flat cells. In the mutant flower, the third whorl organs are made up of conical cells which characterize the petal identity in both the base and tip regions corresponding to the filament and anther region, respectively, of the wild-type stamen (**l**-**n**). Cell morphologies of the carpel are identical in two types of flowers. Cells of the style epidermis are rectangle (**g**, **o**). The stigma epidermis consists of columnar gland cells (**h**, **p**). WT, wild-type; MU, mutant; S-D, dorsal epidermis of the sepal; S-V, ventral epidermis of the sepal; Pet, petal; A-D, dorsal epidermis of the anther; A-V, ventral epidermis of the anther; Fil, filament; Sti, stigma; Sty, style. Bar, 300 μm

To further characterize the organ identity of the third floral whorl of the mutant F_1_ hybrids, we conducted cell morphological analyses of all floral organs in both wild-type and mutant flowers. From wild-type to mutant flowers, no difference in cell morphology was observed in the first, second and fourth whorl floral organs. The sepal of both types is comprised of flat cells in both dorsal and ventral epidermis with dorsal epidermal hairs in the wild-type, but not in the mutant (Fig. 2a, b, i, j). Similarly, all petals are composed of conical cells in both types of flowers (Fig. 2c, k). The carpel is characterized by rectangle cells forming style and columnar gland cells forming stigma, in both wild-type and mutant flowers (Fig. 2g, h, o, p).

In contrast to the unchanged cell morphology of the first, second and fourth whorl floral organs, the cell morphology of the third whorl floral organs is distinctively different between mutant and wild-type flowers. In wild-type flowers, the epidermal cells are flat in filaments and the ventral side of anthers, but reticular in the dorsal side of anthers (Fig. 2d-f). Conversely, in the mutant flower, the epidermis of both the base and tip regions of the petaloid-stamen consists of conical cells, which mimics the wild-type petal cell characteristics, despite some chimeric character in the base region (Fig. 2l-n). The comparative cell morphological analyses further confirm the transformation from stamen to petal identity, i.e. petaloid-stamen, in the third floral whorl of the mutant flower.

### Identification of A-, B-, C-class and *CYC*-like genes

**Fig. 3:**
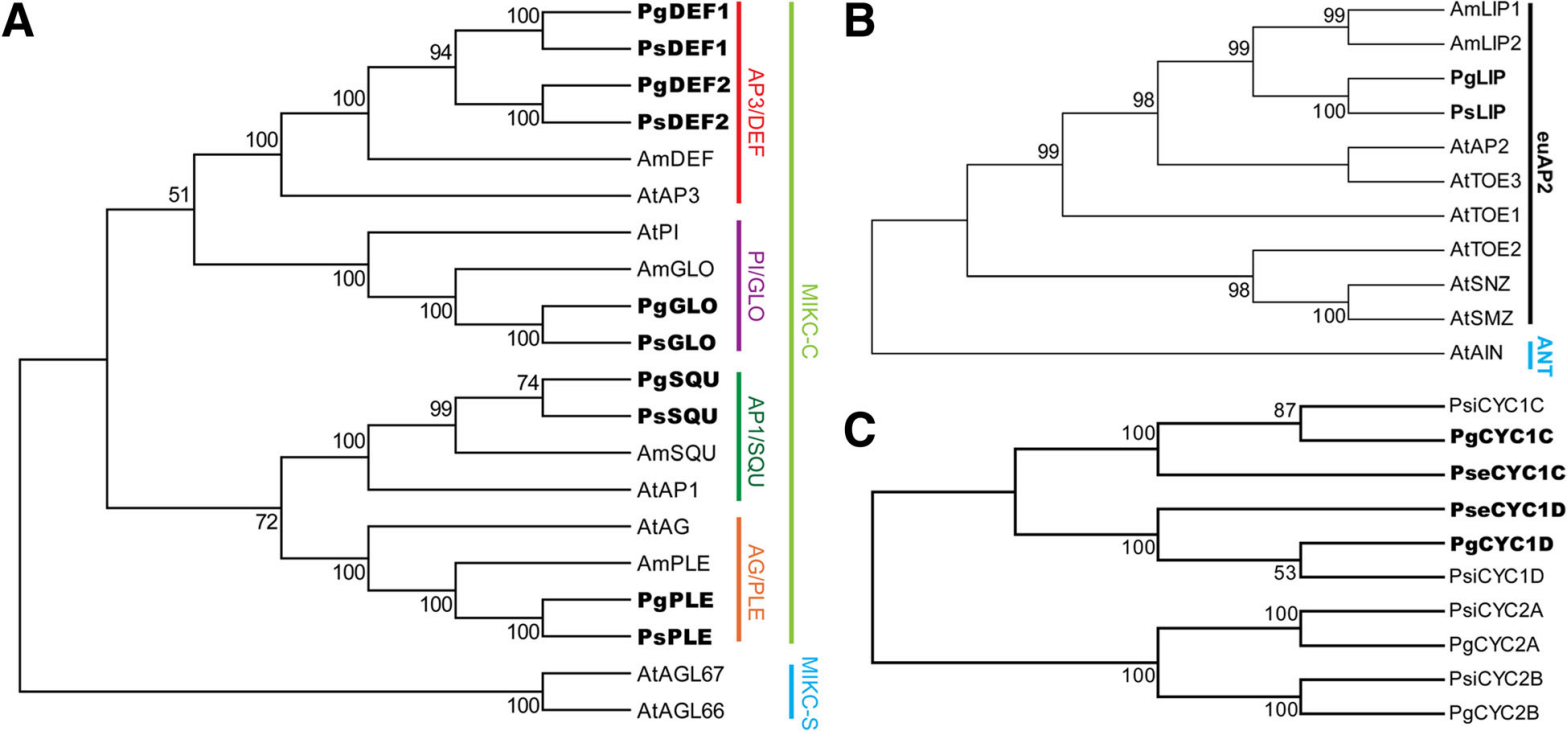
Neighbor-joining tree of A-, B- and C-class MADS-box, AP2/EREBP family and *CYC*-like genes. **a** Tree of A-, B- and C-class MADS-box proteins. Sequences are from *Antirrhinum majus* and *Arabidopsis thaliana*. The tree is calculated based on protein sequences of partial MADS domain and K domain. Neighbor-joining tree shows that *SQU*-, *DEF-*, *GLO-* and *PLE*-like genes amplified in *Petrocosmea glabristoma* and *P. sericea* are well clustered with the corresponding orthologs in *A. majus* and *A. thaliana*, respectively*.* **b** Tree of AP2/EREBP family proteins. The tree is calculated based on protein sequences between the two conserved AP2 domains. AmLIP1 and AmLIP2 are from *A. majus*. Others are from *A. thaliana*. In this tree, PgLIP, PsLIP and AmLIP1/2 form a clade that is further clustered with AtAP2 from *A. thaliana.* **c** Neighbor-joining tree of CYC-like proteins in *Petrocosmea*. All sequences used are from the CYC2 clade TCP proteins. PseCYC1C and PseCYC1D are well clustered with PsiCYC1C/PgCYC1C and PsiCYC1D/PgCYC1D, respectively. The numbers above internal branches give bootstrap probabilities above 50%. Genes isolated in this study are bold. Group names are marked on the right side. Am, *A. majus*; At, *A. thaliana*; Pg, *P*. *glabristoma*; Ps, *P. sericea*, Pse, *P. sericea*; Psi, *P. sinensis*

The MADS-box and *AP2*-like A-, B- and C-class floral organ identity genes were cloned in both two parents and their F_1_ hybrids. Altogether, we cloned one AP1/SQU, one AP2/LIP, two AP3/DEF, one PI/GLO and one AG/PLE genes from each parent and were thus designated as *PgSQU*/*PsSQU*, *PgLIP*/*PsLIP*, *PgDEF1*/*PsDEF1*, *PgDEF2*/*PsDEF2*, *PgGLO*/*PsGLO* and *PgPLE*/*PsPLE* in *P*. *glabristoma* and *P. sericea*, respectively. Phylogenetic reconstruction following sequence alignments showed that the A-class gene *PgSQU*/*PsSQU*, B-class genes *PgDEF1*/*PsDEF1*, *PgDEF2*/*PsDEF2* and *PgGLO*/*PsGLO* as well as the C-class gene *PgPLE*/*PsPLE* were well clustered together with their respective orthologs belonging to the MIKC-C clade MADS-box genes (Fig. 3a, Additional file 1: Figure S2A). Further analyses showed that the two *DEF* paralogs amplified in *Petrocosmea* might come from an ancient duplication (Additional file 1: Figure S2B). The *PgLIP*/*PsLIP* gene was also well grouped with *AtAP2* and *AmLIP1/2* within the euAP2 lineage (Fig. 3b, Additional file 1: Figure S3).

As to *CYC*-like genes, a recent report shows that only *CYC1C* and *CYC1D* are highly expressed in the dorsal petals in *Petrocosmea* (including *P*. *glabristoma* we focused on here), while both *CYC2A* and *CYC2B* show almost undetectable expression signals in all floral organs [35]. Therefore, we here only focused on *CYC1C* and *CYC1D* and their orthologs were isolated from *P. sericea*, respectively. Sequence alignment showed that they had very high sequence similarity to the corresponding orthologs from *P*. *glabristoma* and *P. sinensis* and were well placed in the CYC1C and CYC1D subclade in the Neighbor-joining (NJ) tree, respectively (Fig. 3c, Additional file 1: Figure S4).

### Expression analyses of A-, B- and C-class genes in the parents as well as their wild-type and petaloid-stamen mutant F_1_ hybrid flowers

**Fig. 4:**
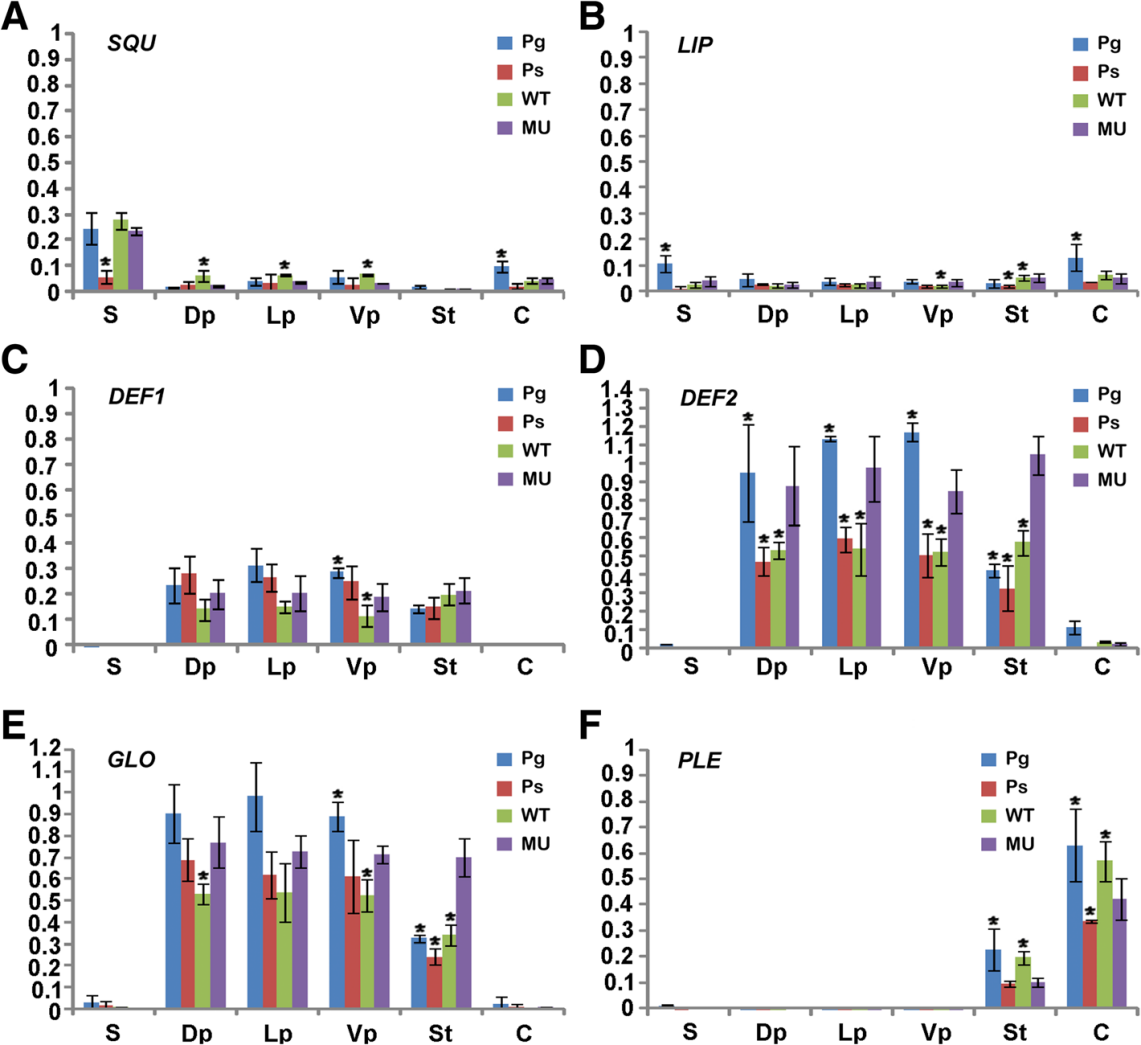
Comparative expression analyses of A-, B- and C-class genes. Real-time PCR were carried out in *Petrocosmea glabristoma*, *P. sericea* and their wild-type and petaloid-stamen mutant F_1_ hybrid flowers. *SQU* genes are expressed in all organs with an equal level in the mutant and wild-type as well as their parental flowers, despite the low expression level in the third floral whorl (**a**). *LIP* genes are universally expressed in all organs in the wild-type and mutant as well as their parental flowers (**b**). The transcripts of B-class genes *DEF1* (**c**), *DEF2* (**d**) and *GLO* (**e**) in both parental and F_1_ hybrid flowers are restricted in the second and third whorls, though slightly expansion into the fourth whorl in *P. glabristoma* flowers. However, the expression levels of both *DEF2* and *GLO* genes are up-regulated in mutant flowers (**d**, **e**). The transcripts of *PLE* genes are restricted in the inner two whorls in both parental and F_1_ hybrid flowers with much lower expression level in the mutant than in the wild-type F_1_ hybrid flowers (**f**). *ACTIN* gene was amplified as an internal control. The values shown (mean ± SD) are the average of three biological replicates with each for three technical repeats. Asterisks indicate that mean values are significantly different between the mutants and their parents as well as their wild-type counterparts (*P* < 0.05). S, sepal; Dp, dorsal petal; Lp, lateral petal; Vp, ventral petal; St, stamen or petaloid-stamen; C, carpel; Pg, *P*. *glabristoma*; Ps, *P. sericea*; WT, wild-type; MU, mutant

To investigate the expression pattern of A-, B- and C-class genes in the parental species *P*. *glabristoma* and *P. sericea* as well as their wild-type and petaloid-stamen mutant F_1_ hybrids, Real-time PCR was performed on cDNAs from each floral organ. In *P*. *glabristoma*, both *SQU* and *LIP* were mainly expressed in sepals and carpels, with low expression level in petals and stamens. In *P. sericea*, both *SQU* and *LIP* were expressed at low level in all organs examined, with nearly undetectable *SQU* expression in stamens (Fig. 4a, b). Similar to the expression pattern of *PgSQU* in *P*. *glabristoma*, *SQU* was mainly expressed in the first, second and fourth whorl floral organs with the highest level in sepals, in both wild-type and mutant F_1_ hybrid flowers (Fig. 4a). *LIP* was universally expressed at low level in all organs of both type F_1_ hybrid flowers, with generally similar expression pattern to their parents (Fig. 4b).

In both parents, B-class genes *DEF1*, *DEF2* and *GLO* were all mainly expressed in the second and third floral whorls, although the transcripts of *DEF2* in *P*. *glabristoma* expanded to the carpel at relatively low expression level (Fig. 4c-e). Further, *DEF2* and *GLO* genes showed an expression level obviously higher in the second than in the third whorl and *DEF1* gene demonstrated an expression level wholly lower than *DEF2* and *GLO* in corresponding organs (Fig. 4c-e). All *DEF1*, *DEF2* and *GLO* transcripts were mainly restricted in the second and third whorls in both types of F_1_ hybrid flowers. *DEF1* showed similar accumulation level in mutant and wild-type F_1_ hybrids as well as their parents (Fig. 4c). However, *DEF2* and *GLO* demonstrated a significantly higher expression level in the mutant than in the wild-type flowers, with their expression level falling between the two parents in the second whorl. Interestingly, the expression level of *DEF2* and *GLO* were about or over two-times higher, respectively, in the mutant than in the wild-type as well as the two parental flowers in the third whorl (Fig. 4d, e).

For the C-class gene *PLE*, its transcripts were specially restricted to the inner two whorls and the expression level was lower in the third than in the fourth whorl in both parents (Fig. 4f). Unexpectedly, the *PLE* expression level is lower in *P. sericea* than in *P*. *glabristoma* in corresponding organs (Fig. 4f). In the wild-type and mutant F_1_ hybrids, *PLE* transcripts were also restricted to the inner two whorls with expression level remarkably lower in the third than in the fourth whorl, similar to that in their parents (Fig. 4f). In the third floral whorl, interestingly, the *PLE* expression level was sharply decreased in the mutant to only half of that in the wild-type F_1_ hybrid flowers (Fig. 4f).

As outlined above, A-, B- and C-class genes had generally similar expression patterns in *P*. *glabristoma* and *P. sericea*. The key difference between the mutant and wild-type F_1_ hybrid flowers is the elevated expression level of the two B-class *DEF2* and *GLO* paralogs and the decreased expression level of the C-class *PLE* in the third floral whorl, i.e. petaloid-stamens, of mutant flowers. In addition, none of the MADS-box gene expression shows a dorso-ventral pattern in both wild-type and mutant flowers (Fig. 4).

### Comparative expression analyses of *CYC-*like genes in wild-type and petaloid-stamen mutant flowers

**Fig. 5:**
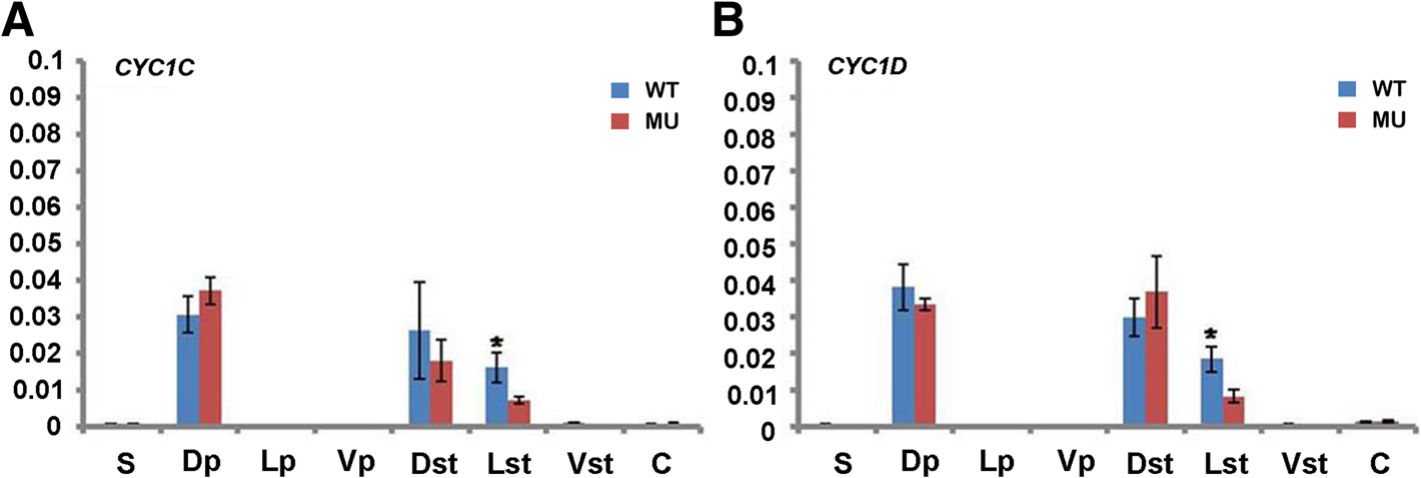
Comparative expression analyses of *CYC*-like genes. Real-time PCR were carried out in wild-type and petaloid-stamen mutant flowers of *Petrocosmea glabristoma* × *Petrocosmea sericea* F_1_ hybrids. *CYC1C* (**a**) and *CYC1D* (**b**) are both highly expressed in dorsal petals as well as dorsal and lateral third floral whorl organs in both the wild-type and mutant flower with a similar expression level. *ACTIN* gene was amplified as an internal control. The values shown (mean ± SD) are the average of three biological replicates with each for three technical repeats. Asterisks indicate that mean values are significantly different between the mutants and their wild-type counterparts (P < 0.05). S, sepal; Dp, dorsal petal; Lp, lateral petal; Vp, ventral petal; Dst, dorsal stamen or petaloid-stamen; Lst, lateral stamen or petaloid-stamen; Vst, ventral stamen or petaloid-stamen; C, carpel; WT, wild-type; MU, mutant

To investigate the activity of *CYC*-like genes, comparative expression analyses were conducted in wild-type and mutant flowers. In the wild-type flower, both *CYC1C* and *CYC1D* were highly expressed in dorsal petals as well as dorsal and lateral staminodes, while their transcript signals were undetectable in other organs examined (Fig. 5a, b). These results agree with the data previously reported in Gesneriaceae [35, 38, 39]. In the mutant flower, both *CYC1C* and *CYC1D* were also specially expressed in the dorsal petals in the second whorl, dorsal and lateral petaloid-stamens in the third whorl, undetectable in the ventral organs of both second and third whorls. The lower expression level in the lateral petaloid-stamen relative to that in the wild-type lateral staminodes is correlated with the increased size of the lateral petaloid-stamen (Figs. 1d, f and 5a, b). Thus, the expression patterns of *CYC1C* and *CYC1D* remain unchanged from wild-type to mutant flowers, consistent with the unchanged zygomorphic phenotype in the mutant flower.

### ASE analyses of *DEF2*, *GLO* and *PLE*

**Fig. 6:**
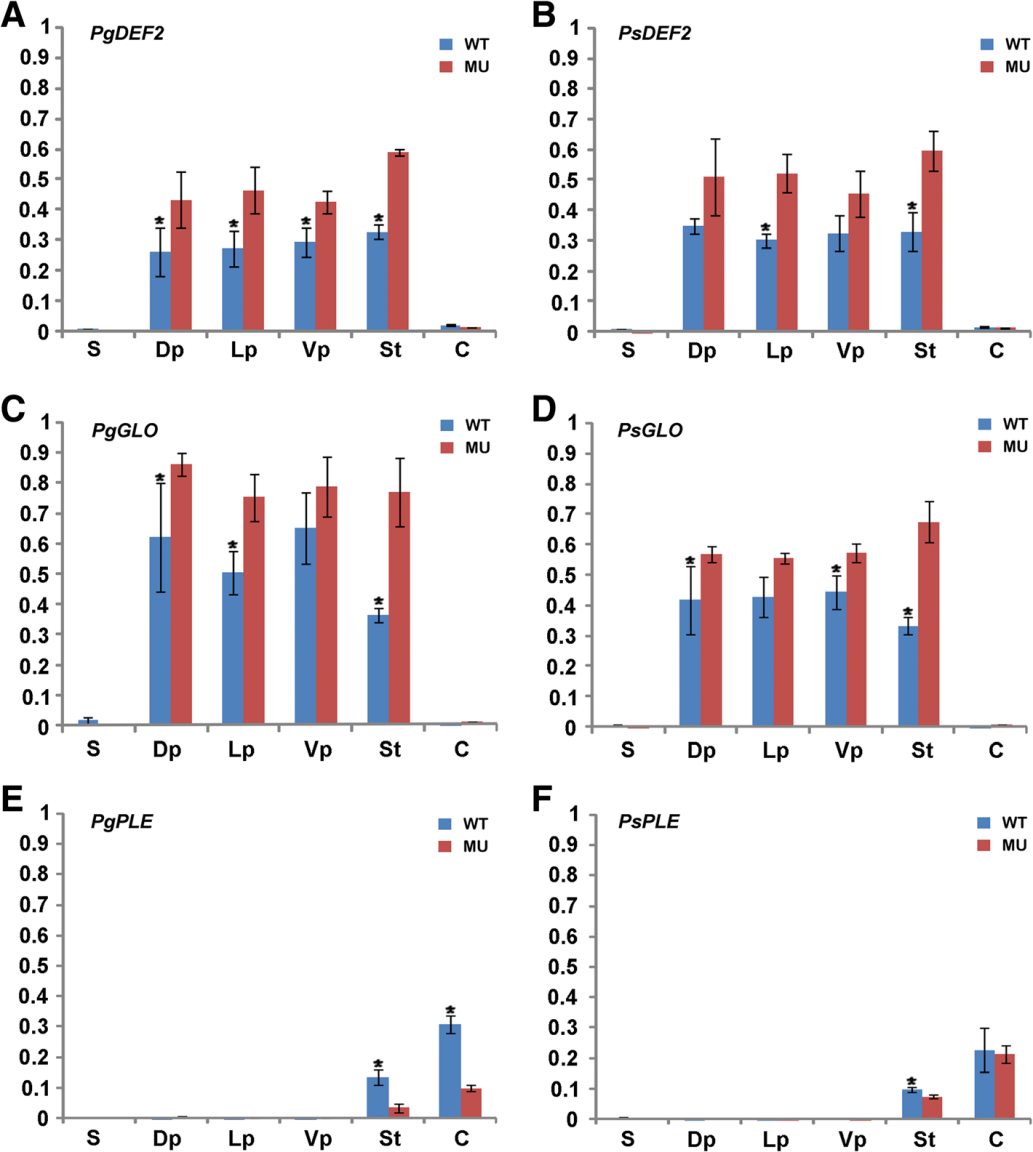
Allele-specific expression analyses of *DEF2*, *GLO* and *PLE* between wild-type and mutant F_1_ hybrids. **a** Expression pattern of *PgDEF2* allele in wild-type and mutant F_1_ hybrids. **b** Expression pattern of *PsDEF2* allele in wild-type and mutant F_1_ hybrids. **c** Expression pattern of *PgGLO* allele in wild-type and mutant F_1_ hybrids. **d** Expression pattern of *PsGLO* allele in wild-type and mutant F_1_ hybrids. **e** Expression pattern of *PgPLE* allele in wild-type and mutant F_1_ hybrids. **f** Expression pattern of *PsPLE* allele in wild-type and mutant F_1_ hybrids. *ACTIN* gene was amplified as an internal control. The values shown (mean ± SD) are the average of three biological replicates with each for three technical repeats. Asterisks indicate that mean values are significantly different between the mutants and their wild-type counterparts (P < 0.05). S, sepal; Dp, dorsal petal; Lp, lateral petal; Vp, ventral petal; St, stamen or petaloid-stamen; C, carpel; WT, wild-type; MU, mutant

Given that *DEF2*, *GLO* and *PLE* were differently expressed between wild-type and mutant F_1_ hybrid flowers, we further conducted ASE analyses to determine which allele account for the expression differentiation of each gene and make out the differential contribution of each parent. First, we identified the SNP sites for each orthologous pair by sequencing the PCR products of *DEF2*, *GLO* and *PLE* amplified from two parents for three different replicates, respectively, which were further confirmed by genotyping both the wild-type and mutant F_1_ hybrids. Through sequencing three independent individuals of the two parents and their F_1_ hybrids, respectively, we confirm that the allelic difference between parents is fixed and the sequences amplified in mutant F_1_ hybrids are the same as their wild-type counterparts (Additional file 1: Figure S5). For *DEF2* and *GLO*, the two alleles of each gene had almost equal increase degree in expression levels in corresponding organs from wild-type to mutant F_1_ hybrids. In the third whorl, the expression level was about two-times higher for the two *DEF2* alleles and over two-times higher for the two *GLO* alleles in mutant than in wild-type flowers, reminiscent of the differential expression level of *DEF2* and *GLO*, respectively, between wild-type and mutant flowers (Fig. 6a-d). Namely, for *DEF2* and *GLO*, both the two parental alleles of each gene contribute equally to the elevated expression level in the mutant flower. In contrast, the two *PLE* alleles *PgPLE* and *PsPLE* had remarkably differential expression level in both third and fourth whorls. From wild-type to mutant flowers, the *PgPLE* allele demonstrated a significantly decreased expression level to about a quarter, while the *PsPLE* allele showed a slightly reduced expression level (Fig. 6e, f). Apparently, the *PgPLE* allele, i.e. the parent *P*. *glabristoma*, has dominant contribution to the decreased *PLE* expression level in mutant flowers.

### Phenotypic variation in flowers of *Petrocosmea* hybrids

In addition to above experiments, we also conducted a series of crosses between *P*. *glabristoma* and other related species in *Petrocosmea*. We found some dorsalized tetramerous or pentamerous flowers besides the petaloid-stamen mutant in F_1_ hybrids of *P*. *glabristoma* × *P. sericea* (Additional file 1: Figure S6A, B). In *P. nervosa* × *P*. *glabristoma*, the wild-type hybrid was similar to *P. nervosa* in each petal shape,but similar to *P*. *glabristoma* in the angle between each two petals (Fig. 1a, Additional file 1: Figure S6C, D). We found three mutant flowers exhibiting severe phenotype of petaloid-stamen among 187 flowers examined (mutation rate: 1.6%) (Additional file 1: Figure S6E). In *P. oblata* × *P*. *glabristoma*, the wild-type hybrid was very similar to *P. oblata* (Additional file 1: Figure S6F, G). We observed two types of eight mutants among 224 flowers (mutation rate: 3.6%), exhibiting four dorsal petals plus two fertile ventral stamens and dorsalized hexamerous petals with stamens aborted, respectively (Additional file 1: Figure S6H, I). In *P. qinlingensis* × *P*. *glabristoma* hybrids, the wild-type flower is morphological intermediate between two parents. About 30 dorsalized tetramerous or pentamerous flowers with stamens aborted were observed out of 400 flowers checked (mutation rate: 7.5%, Additional file 1: Figure S6L, M).

**Fig. 7:**
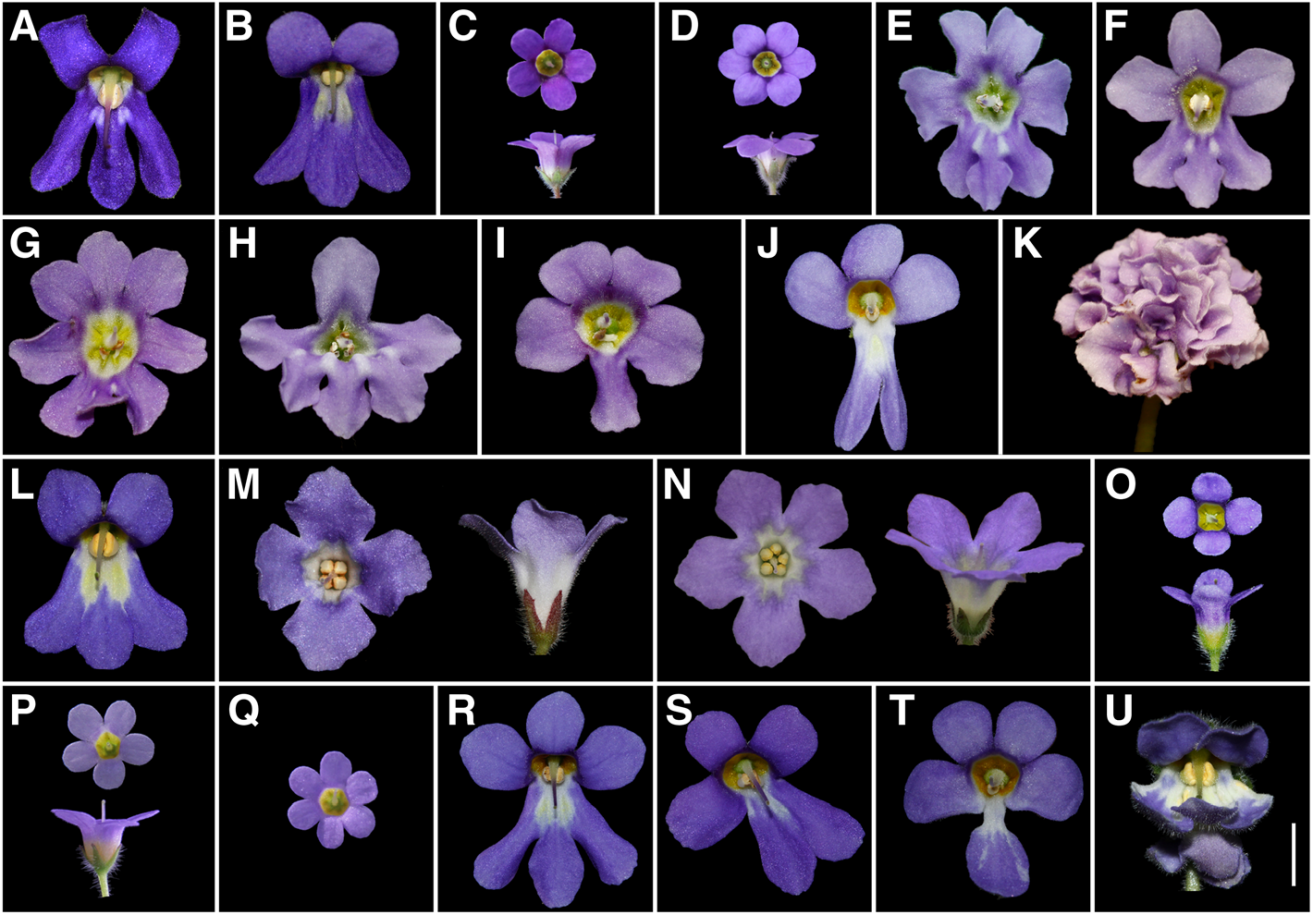
Wild-type and mutant floral morphology in the hybrids of *Petrocosmea glabristoma* and *P. sinensis*. **a** flower of *P. sinensis*; **b**-**k** flowers of F_1_ hybrids of *P*. *glabristoma* × *P. sinensis*. **b** Wild-type flower of F_1_ hybrids; **c**, **d** dorsalized pentamerous (**c**) and hexamerous (**d**) flowers in face and lateral view; **e**-**g** flower with four (**e**), three (**f**) and five (**g**) dorsal petals; (**h**) flower with one large dorsal petal, two lateral petals and three ventral petals; **i**-**j** flower with four dorsal petals and one ventral petal (**i**), three dorsal petals and two ventral petals (**j**) (lateral petals are missing); **k** several flowers transformed to a “head-like” inflorescences, each floret having only uncertain number of petals and sepals with complete missing of stamen and pistil (lateral view). **l**-**u** flowers of F_1_ hybrids of *P. sinensis* × *P*. *glabristoma*. **l** wild-type flower of F_1_ hybrids; **m**, **n** ventralized tetramerous (**m**) and pentamerous (**n**) flowers in face and lateral view; **o**-**q** dorsalized tetramerous (**o**), pentamerous (**p**), hexamerous (**q**) flowers in face and lateral view; **r** flower with three dorsal petals; **s** flowers with the left lateral petal reduced in size, ventral petal right-inclined plus two fertile stamens; **t** flower with four dorsal petals and one ventral petal; **u** flower with additional petals between lower lips and sepals. Bar, 0.5 cm

In *P*. *glabristoma* × *P. sinensis*, the wild-type hybrid generally exhibited an intermediate phenotype between two parents (Figs. 1a and 7a, b). Five types of about 125 mutants were observed out of more than 1500 flowers checked (mutation rate: 8.3%). They exhibit (1) dorsalized petals with stamens aborted (Fig. 7c, d); (2) three to five dorsal petals with two fertile ventral stamens (Fig. 7e-g); (3) one large dorsal petal and three ventral petals with four protuberances at the junction between ventral and lateral petals with one dorsal and two ventral stamens fertile and two lateral stamens sterile (Fig. 7h); (4) four dorsal and one ventral petals or three dorsal and two ventral petals, with only one fertile ventral stamen (Fig. 7i-j); (5) ‘head-like’ inflorescence formed by several abnormal flowers (Fig. 7k).

In *P. sinensis* × *P*. *glabristoma*, the wild-type hybrid was almost identical to the one of their reciprocal hybrids (Fig. 7b, l). Six types of about 146 mutants were observed out of more than 1600 flowers checked (mutation rate: 9.1%). They demonstrate (1) ventralized petals with corolla-tube lengthened and all stamens fertile (Fig. 7m, n); (2) dorsalized petals with elongated corolla tube and wholly sterile stamen (Fig. 7o-q); (3) three dorsal petals and two fertile stamens (Fig. 7r); (4) the left lateral petal reduced in size, ventral petal right-inclined plus two fertile stamens (Fig. 7s); (5) four dorsal and one ventral petal plus one fertile stamen (Fig. 7t); (6) additional petals developed between the lower lip and sepals (Fig. 7u).

## Discussion

### Consideration of the expression of A-, B- and C-class genes in the wild-type hybrid and their parental *P*. *glabristoma* and *P. sericea*

The transcripts of the A-class gene *SQU* in wild-type *Petrocosmea* hybrid flowers spread through all floral whorls despite nearly undetectable in stamens, which is similar to the expression pattern of *PgSQU*/*PsSQU* in their parental species and its orthologs in Asteridae, i.e. *SQU* in *A. majus* and *FBP26* in *Petunia* that are expressed in sepals, petals, carpels and the early developing stamens [6, 40–42]. These similar expression patterns are consistent with the functional activity of *SQU*-like genes. For example, the mutation of *SQU* in *A. majus* results in the development of shoots at positions of flowers, but the flower organ identity are normally developed [40]. The down-regulation of *FBP26* blocks the transition from vegetative to reproductive development [41]. *SQU*-like genes might have more role in floral meristem identity and activation of B- and C-class genes than in sepal and petal identity as suggested previously [13, 43–46]. In contrast, the spatial expression pattern of *SQU* is distinctively different from its orthologs in Rosidae, i.e. *AP1* in *A. thaliana* and *PEAM4* in pea that are mainly expressed in sepals and petals, functionally responsible for the sepal and petal identity [4, 47]. Another A-class gene *LIP*, a member of AP2/EREBP transcription factor family, shows a similar expression pattern in wild-type *Petrocosmea* hybrids with *PgLIP*/*PsLIP* in the parental species and *AP2* in *A. thaliana* and *LIP1/2* in *A. majus* that are transcribed in all four floral whorls, except for *LIP1/2* losing expressional signals in the later stage sepals [9, 48]. Unfortunately, little has been known about *AP2/LIP*-like genes outside the two model species.

In both *Petrocosmea* wild-type F_1_ hybrid and their parental flowers, the transcripts of B-class paralogs *DEF1*/*2*, *GLO* and C-class *PLE* are restricted in the second/third and third/fourth whorls, respectively, which fit perfectly into the frame of classical ABC model [1, 49]. The B-class genes are conserved in expression range in eudicots, despite a slight expansion to the fourth whorl sometimes which was also observed in the female parent *P*. *glabristoma* (Fig. 4d) [5, 7, 8, 50–52]. The C-class gene *PLE* in both parental and wild-type F_1_ hybrid flowers displays a similar expression pattern with *PLE* in *A. majus*, *AG* in *A. thaliana* and *pMADS3*, *FBP6* in *Petunia* [53–55]. However, the expression level of *PLE* in *Petrocosmea* is higher in the fourth whorl than in the third whorl, similar to that of *FBP6* in *Petunia* [55]. As outlined above, our results strengthen the argument that the A function genes in controlling the sepal and petal identity might be not conserved among major clades in angiosperms (also see review by [56]), and are indicative of redefinition of the A function in floral development as previously suggested based on functional evidence [13, 45]. The results herein, along with previous reports in gene function and expression, suggest that B- and C-class genes have conserved functions in determining the petal/stamen and stamen/gynoecium identities, respectively, in eudicots. In addition, compared to the expression level in the third whorl, the higher level of C-class transcripts, especially *PLE* herein, in the center whorl may hint at a conserved binary role of C-class gene in determining both the carpel identity and flower determination in the fourth whorl.

### Interpretation of the petaloid-stamen mutant

In the petaloid-stamen mutant, A-, B- and C-class genes are all expressed in accordance with their respective floral regions in the wild-type F_1_ hybrid. However, the expression levels of B-class *DEF2* and *GLO* are remarkably increased and the transcripts of C-class *PLE* are sharply reduced in the petaloid-stamen of mutant flowers compared to that in the stamen of wild-type flowers. These expressional changes are strongly correlated with the phenotype of petaloid-stamen with conical cell type which characterizes the petal identity of wild-type F_1_ hybrid flowers as well as flowers in other groups [57]. The petaloid-stamen in the F_1_ hybrid mutant flowers might be induced by the excessive expression of B-class genes and less transcripts of the C-class gene. In many groups, including tobacco, rape, *Petunia*, stem mustard and wheat, the transcriptional decrease or mutation of the B-class genes in third whorl usually produces sterile stamen or carpelloid structure [58–62]. In orchids, the increased level of clade 3 and 4 *DEF*-like genes leads to the transformation of the two inner lateral tepals into the labellum to form a peloria [63, 64]. Correspondingly, it has been widely reported from a large number of groups, such as *Petunia*, stem mustard, torenia, Kurume azaleas, rose and lily, as well as model species *A. thaliana* and *A. majus*, that the petaloid-stamen is usually consequent upon down-regulation or silence of C-class genes in the third floral whorl [27, 30, 32, 33, 62, 65–67].

In the ABC model, the stamen identity is characterized by the function and interaction of B- and C-class genes [68]. The above evidence indicates that the up-regulation or down-regulation (or silence) of either B-class or C-class genes or both of them happened simultaneously in fact may break the B-C interaction balance in the third floral whorl. The appropriate amount of B- and C-class gene transcripts, i.e. a dosage balance between B- and C-class genes, could be necessary to maintain the stamen identity. The balance of dosage-dependent regulators in a system ensures that each gene affects one aspect of the phenotype, and variation in the dosage of individual components can affect the function of the whole [69]. For example, *AGL24*, a MADS-box gene, is a dosage-dependent promoter of flowering. The loss or reduction of *AGL24* activity results in late flowering while its over-expression causes precocious flowering [70]. We herein demonstrate that a dosage imbalance between B- and C-class genes, i.e. up-regulation of B-class and down-regulation of C-class genes, gives rise to a petaloid-stamen mutant.

Addressing why the B-class genes *DEF2* and *GLO* are excessively expressed while the C-class gene *PLE* is down-regulated in the petaloid-stamens of the mutant F_1_ hybrid, our ASE analyses further show that each two alleles of the B-class genes, i.e. *PgDEF2*/*PsDEF2* and *PgGLO*/*PsGLO*, demonstrate similar degrees of expression increase while the two C-class gene alleles *PgPLE/PsPLE* exhibit different degrees of expression decrease from wild-type to petaloid-stamen flowers (Fig. 6a-d). The similar increase degrees of each two allele expression level of B-class genes in the mutant suggests that these expression changes tend to be caused by changes in *trans*-regulation, such as common upstream transcription factors or cytoplasmic environment. However, the different decrease degrees of the two *PLE* allele expression level might be attributed to changes in *cis*-regulation in *PgPLE* regulatory region, such as DNA methylation and histone modification, or even nucleotide substitution at putative DNA binding sites (Fig. 6e, f). In addition, the *PgPLE* allele should have major contribution to the decreased *PLE* expression in the petaloid-stamen because of the *PgPLE* allele remaining almost unchanged expression level in the wild-type F_1_ hybrid while excessively reducing transcripts in the petaloid-stamen mutant (Fig. 6e). In diploid organisms, *cis*-regulatory mutations can be defined as those that change gene expression in an allele-specific manner, while *trans*-regulatory mutations influence gene expression in a diffusible manner, such as mutations in transcription factors [71]. A series of studies in *A. majus* and poppy provide strong evidence that B- and C-class genes have antagonistic effect on determinate development in the fourth whorl [5, 72, 73]. It is also likely that the B- and C-class genes may be antagonistic to each other in the third floral whorl in *Petrocosmea*. The *PLE* products may act as an upstream *trans*-regulatory factor repressing the B-class *DEF2* and *GLO* expression in the third whorl whose reduction might have led to the increase of *DEF2* and *GLO* transcripts. It would be interesting to conduct functional investigation to confirm whether there is an antagonistic relationship between B- and C-class genes, especially if the C-class PLE represses B-class gene activities in the third floral whorl.

**Fig. 8:**
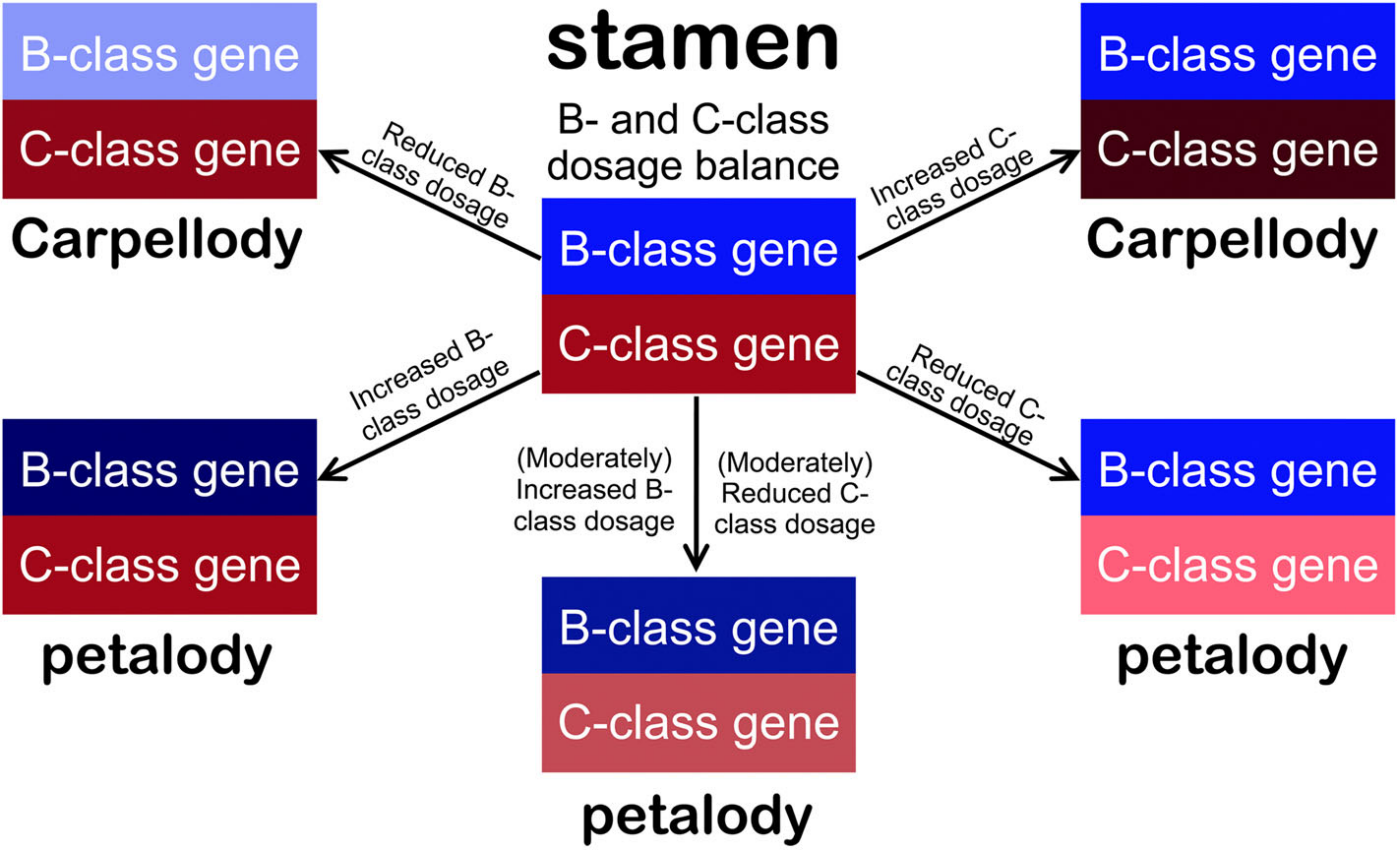
Diagram of dosage imbalance of B-/C-class genes associated with third floral whorl organ identities. Blue rectangles represent the B-class gene products and red rectangles represent the C-class gene products. The color deepness is positively correlated with the dosage of corresponding gene products. The balanced dosage between B- and C-class gene products produces the normal stamen in the third floral whorl (the center). When either the B-class gene products reduce or the C-class gene products increase, the stamen transforms into carpellody. However, petalody can be induced by either the increased B-class gene products or the reduced C-class gene products or both happened simultaneously

Our results together with previous data show that the mechanism underlying the imbalance between B- and C-class genes is quite divergent in the third floral whorl, which make stamens to develop into petalody or carpellody. Taking all above facts into consideration, we here try to provide a model for the dosage-dependent interaction of B- and C-class genes associated with organ identity changes in the third floral whorl. The model suggests that the stamen identity is maintained based on a balance of B- and C-class activities. Once the balance is broken, the homeotic organs would develop in the third whorl. The excessive accumulation of B-class products or the decrease of C-class products or both of them happened simultaneously would result in stamens transformed to petaloid organs. Meanwhile, the decreased B-class or increased C-class products may cause stamens shifted to carpelloid organs (Fig. 8). However, these dynamic interactions of B- and C-class genes and their roles in the floral development of the third floral whorl are hypothesized only based on limited data herein. Further evidence in gene expression, genetic and functional studies is clearly needed before the interesting question can be resolved.

### Genetic relationship between *CYC*-like TCP genes and components of ABC model

The final form of a normal flower often involves two major elements, i.e. the organ types determined by organ identity genes functioning in frame of the ABC model, and the flower architecture mainly controlled by floral symmetry genes in which *CYC*-like TCP genes act as major controlling factors in eudicots. Therefore, the genetic relationship between organ identity genes and *CYC*-like genes has increasingly become an interesting question in floral morphological evolution. To further address this question, we carry out comparative expression analyses of *CYC1C* and *CYC1D* in wild-type and petaloid-stamen mutant F_1_ hybrid flowers. Our results show that the dorso-ventral expression patterns of both *CYC1C* and *CYC1D* in the petaloid-stamen mutant are almost identical to those in wild-type flowers. This is consistent with the unchanged floral zygomorphy in the mutant. The findings herein indicate that *CYC*-like genes and components of the ABC model may act independently in shaping the flower form in *Petrocosmea*. Similarly, it has been reported in Commelinaceae that *CYC*-like and B-class genes are independently recruited to control zygomorphy at early and late stage concluded from comparative expression analyses of *CYC*-like and B-class genes among actinomorphy and distinct zygomorphy species [74]. Functional study using transgenic technology in *Torenia fournieri* of B-class genes also asserts that the altered organ identity resulting from the ectopic or repressed B function cannot induce the change in symmetry at a certain whorl [75]. It would be plausible that the genetic pathway of *CYC*-like genes in controlling floral zygomorphy is in parallel with the floral organ identity pathway. In some cases, the maintenance of *CYC*-like gene activity in late stage may depend on B-class gene products as in *A. majus* [72]. Further broad sampling with functional and regulatory investigation between *CYC*-like genes and components of ABC model would shed new light on such interesting question.

### Significance of the F_1_ hybrid extreme variation in *Petrocosmea*

It has been increasingly recognized that hybridization, especially interspecific hybridization, is a catalyst not only for speciation but also for major evolutionary innovation [76]. Interspecific hybridization upon invasion of new environments usually facilitates rapid adaptive radiation, which would be maintained and prolonged by hybrid swarms of related species that further increase variation to form the basis for more divergence, such as the Darwin’s finch radiation [77]. Our results show that the F_1_ hybrids of *P*. *glabristoma* with other species in *Petrocosmea* display predominantly additive or complementary patterns of morphological characters, especially in corolla shape and structure. However, even though these mosaic and intermediate characters are predominant in F_1_ hybrids, there are considerable numbers of flowers that display extremely deviations or transgressive phenotypes in the F_1_ hybrids. Among them, there are some identical to the natural variants or laboratory mutants such as the typical dorsalized or ventralized flowers reminiscent of floral mutants consequent upon ectopic or loss of expression of the *CYC* gene in *Antirrhinunm* [78] and the petaloid-stamen in third whorl as mentioned above. In a review of 46 studies exploring morphological character expression in hybrids, Rieseberg and Ellstrand [79] pointed out that there is a surprisingly high proportion (10.2%) of F_1_ individual hybrids exhibiting extreme or novel characters and that there are even higher proportions of extreme characters displayed by later generation hybrids (30.6%). Our results together with previous reports indicate that the extremely deviated phenotypes in F_1_ hybrids may represent a sign that remarkably higher frequency of extreme or novel morphological characters would occur in later generation hybrids, i.e. ‘few F_1_s, but numerous later generation hybrids’ as stressed by Rieseberg and Ellstrand [79].

Interspecific hybridization upon interactions among loci with alleles having opposing effect on phenotypes within each parent usually brings about transgressive phenotypes that exceed the phenotypic range of the parental species [80, 81]. In addition, hybridization among related species and divergent populations might lead to elevated mutation rate in hybrids. This is evident in some cases of adaptive radiation, such as the accelerated rates of regulatory gene evolution in the adaptive radiation of Hawaiian silversword alliance descended from an interspecific hybridization [82]. These increased mutation rates upon interspecific hybridization might be due to genetic heterozygosity, heterosis and de novo chromosomal rearrangements that may be directly linked to methylation changes and transposable element derepression [83], or even have genome-wide effects. Rather than the traditional view of an abnormal phenomenon without evolutionary significance, recent theoretical and empirical researches have consistently suggested and confirmed that the extreme hybrids in interspecific hybridizations, usually called ‘hopeful monsters’, represent a common biological feature, which not only have great potential to produce novel phenotypes but also might be an important mechanism firing sudden bursts of variation for rapid adaptive radiation, especially in unoccupied ecological niches or heterogeneous environments [76, 77, 81, 84]. Even though hybridization is frequently performed in the varieties of crops and economic plants, it is still rare to conduct a series of hybridizations in a group of wild plants. Our results provide fresh insights into the role of extreme hybrids in evolution that interspecific hybridization may provide the raw materials for selection acting on and have a major contribution to elevated genetic variability and increased rates of phenotypic evolution.

## Conclusions

To our knowledge, it is the first report that a dosage imbalance between B- and C-class genes is associated with a petaloid-stamen mutant, indicating that the function of B- and C-class genes in controlling stamen identity may depend on an exact dosage balance of their products. Accordingly, we suggest a model for the dosage-dependent interaction of B- and C-class genes associated with organ identity changes in the third floral whorl. In addition, our results are suggestive of a mechanism of petaloid-stamen development through a regulatory modification of one allele in hybrids that effects the change of both B- and C-class gene activities. The genes controlling floral symmetry and component of ABC model regulating floral organ identity might act in parallel genetic pathways. In addition, the hybridization between *P*. *glabristoma* and related species provide fresh evidence that interspecific hybridization likely produces extreme deviants, which represents an important mechanism generating evolutionary innovation and triggering biological diversification. For the interaction of B- and C-class genes, it would be interesting to clarify the regulatory mechanism underlying the dosage imbalance between them, which would shed critical light on establishing a frame reflecting the causal relationship between B- and C-class gene activities and stamen whorl morphologies in angiosperms.

## Additional files

Additional file 1: **Figure S1.** The plants of *Petrocosmea glabristoma* (♀), *P. sericea* (♂) and their F_1_ hybrids. **Figure S2.** Sequence alignment of the MADS-box proteins and Neighbor-joining tree of *DEF*-like genes. **Figure S3.** Sequence alignment of AP2-like proteins. **Figure S4.** Sequence alignment of PseCYC1C/D and PgCYC1C/D with other related proteins. **Figure S5.** SNP identification of *DEF2*, *GLO* and *PLE* genes in *Petrocosmea glabristoma* and *P. sericea*. **Figure S6.** Morphology of wild-type and mutant flowers in the hybrids of *Petrocosmea glabristoma* crossed with other *Petrocosmea* species. **Table S1.** Primers used for gene isolation in this study. **Table S2.** Primers used for Real-time PCR in this study. **Table S3.** Primers used for allele-specific Real-time PCR in this study. (DOC 2903 kb)

Additional file 2: Sequences isolated in this article. (TXT 9 kb)

## Abbreviations

ASE: Allele-specific expression
IBCAS: Institute of Botany, Chinese Academy of Sciences
MS: Murashige and Skoog
NCBI: National Center for Biotechnology Information
NJ: Neighbor-joining
RT-PCR: Reverse transcription PCR
SNP: Single nucleotide polymorphism
