# Supplementary material for: Dosage imbalance of B- and C-class genes causes petaloid-stamen relating to F1 hybrid variation
Source: BMC Plant Biol. 2018 Dec 7;18:341. doi: 10.1186/s12870-018-1562-4 (PMC6286610; doi:10.1186/s12870-018-1562-4)
Supplement: Supplementary file 1 — Figure S1. The plants of Petrocosmea glabristoma (♀), P. sericea (♂) and their F1 hybrids. Figure S2. Sequence alignment of the MADS-box proteins and Neighbor-joining tree of DEF-like genes. Figure S3. Sequence alignment of AP2-like proteins. Figure S4. Sequence alignment of PseCYC1C/D and PgCYC1C/D with other related proteins. Figure S5. SNP identification of DEF2, GLO and PLE genes in Petrocosmea glabristoma and P. sericea. Figure S6. Morphology of wild-type and mutant flowers in the hybrids of Petrocosmea glabristoma crossed with other Petrocosmea species. Table S1. Primers used for gene isolation in this study. Table S2. Primers used for Real-time PCR in this study. Table S3. Primers used for allele-specific Real-time PCR in this study. (DOC 2903 kb) [file 12870_2018_1562_MOESM1_ESM.doc]

**Dosage Imbalance of B- and C-class Genes Causes Petaloid-Stamen Relating to F_1_ Hybrid Variation**

Jing Liu^1,2 #^, Chao-Qun Li^1,2 #^, Yang Dong^1^, Xia Yang^1^, Yin-Zheng Wang^1,2^ *

* **Correspondence:**

Corresponding Author: Yin-Zheng Wang

E-mail address: [wangyz@ibcas.ac.cn](mailto:wangyz@ibcas.ac.cn)

^#^ The two authors contribute equally to this work.

^1^ State Key Laboratory of Systematic and Evolutionary Botany, Institute of Botany, Chinese Academy of Sciences, Beijing 100093, China.

^2^ University of Chinese Academy of Sciences, Beijing 100049, China.


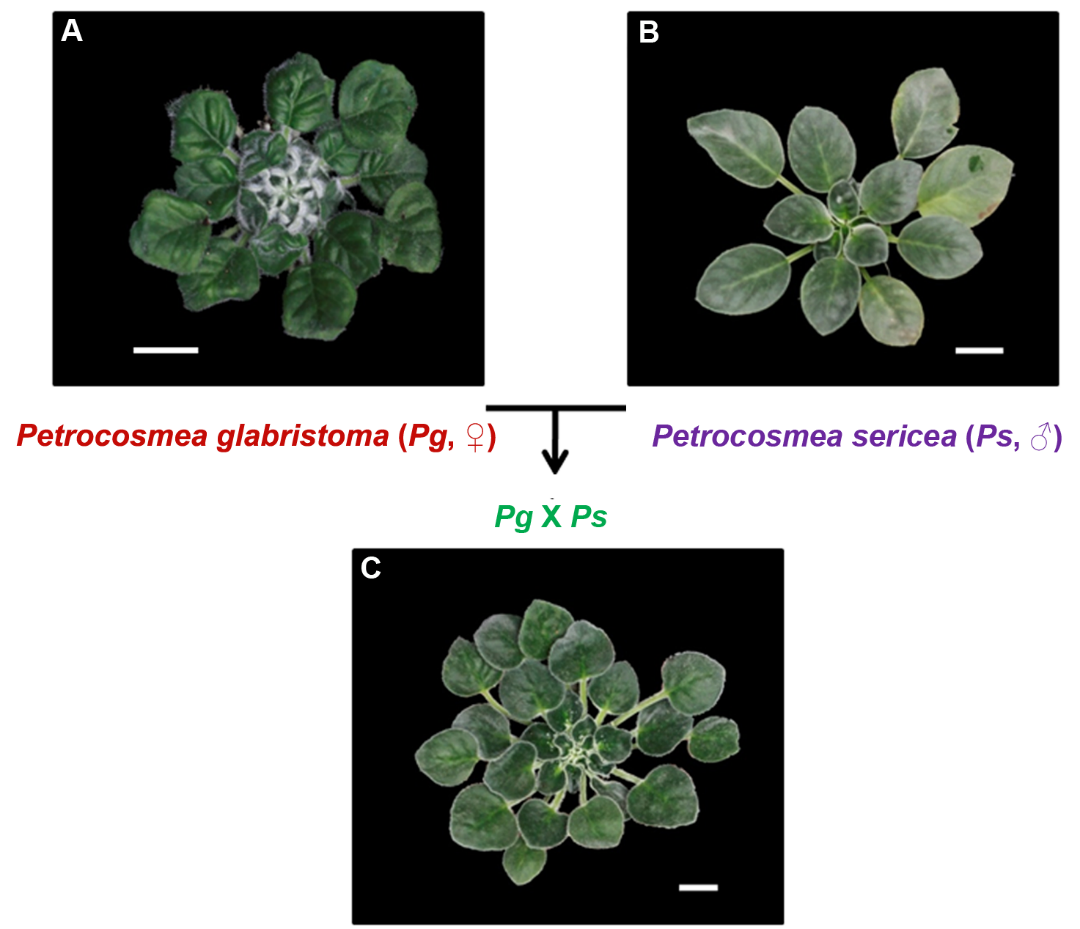


**Figure S1****.** The plants of *Petrocosmea glabristoma* (♀), *P*. *sericea* (♂) and their F_1_ hybrids. *P*. *glabristoma* (A) and *P*. *sericea* (B) are similar in vegetative trait with rosette habit, though some differences in detailed leaf shape. The F_1_ hybrids (C) show similar vegetative features to both parents. Bar, 2cm.


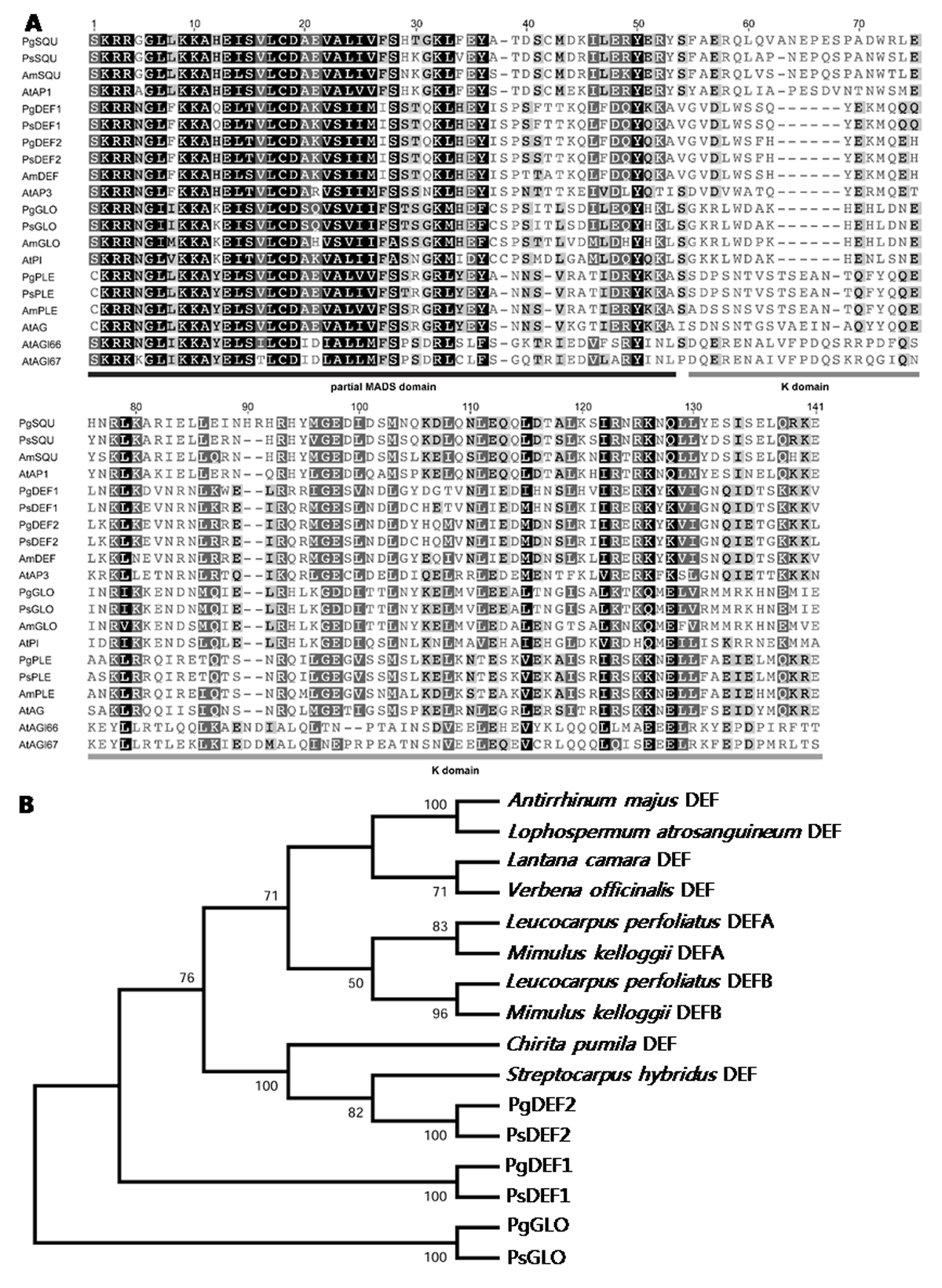
**Figure S2.** Sequence alignment of the MADS-box proteins and Neighbor-joining tree of *DEF*-like genes. Sequences in A are from *Petrocosmea glabristoma* (Pg), *P*. *sericea* (Ps), *Antirrhinum majus* (Am) and *Arabidopsis thaliana* (At). A hyphen represents a gap inserted to optimize alignment. The conserved MADS and K domains are underlined by the black and grey lines, respectively. Sequences in B are from Gesneriaceae and its Lamiales relatives. The numbers above internal branches give bootstrap probabilities above 50%. Pg, *P. glabristoma*; Ps, *P*. *sericea*.


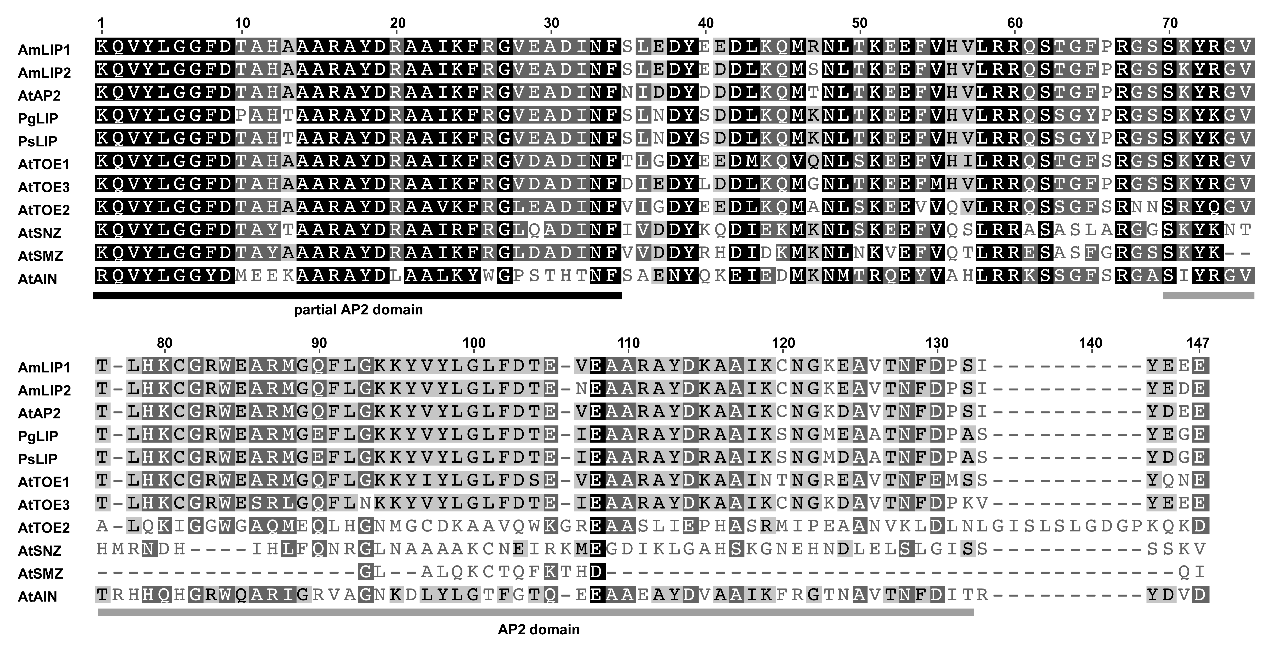


**Figure S3.** Sequence alignment of AP2-like proteins. Sequences are from *Petrocosmea glabristoma* (Pg), *P*. *sericea* (Ps), *Antirrhinum majus* (Am) and *Arabidopsis thaliana* (At). A hyphen represents a gap inserted to optimize alignment. The sequences underlined indicate the two conserved AP2 domains.


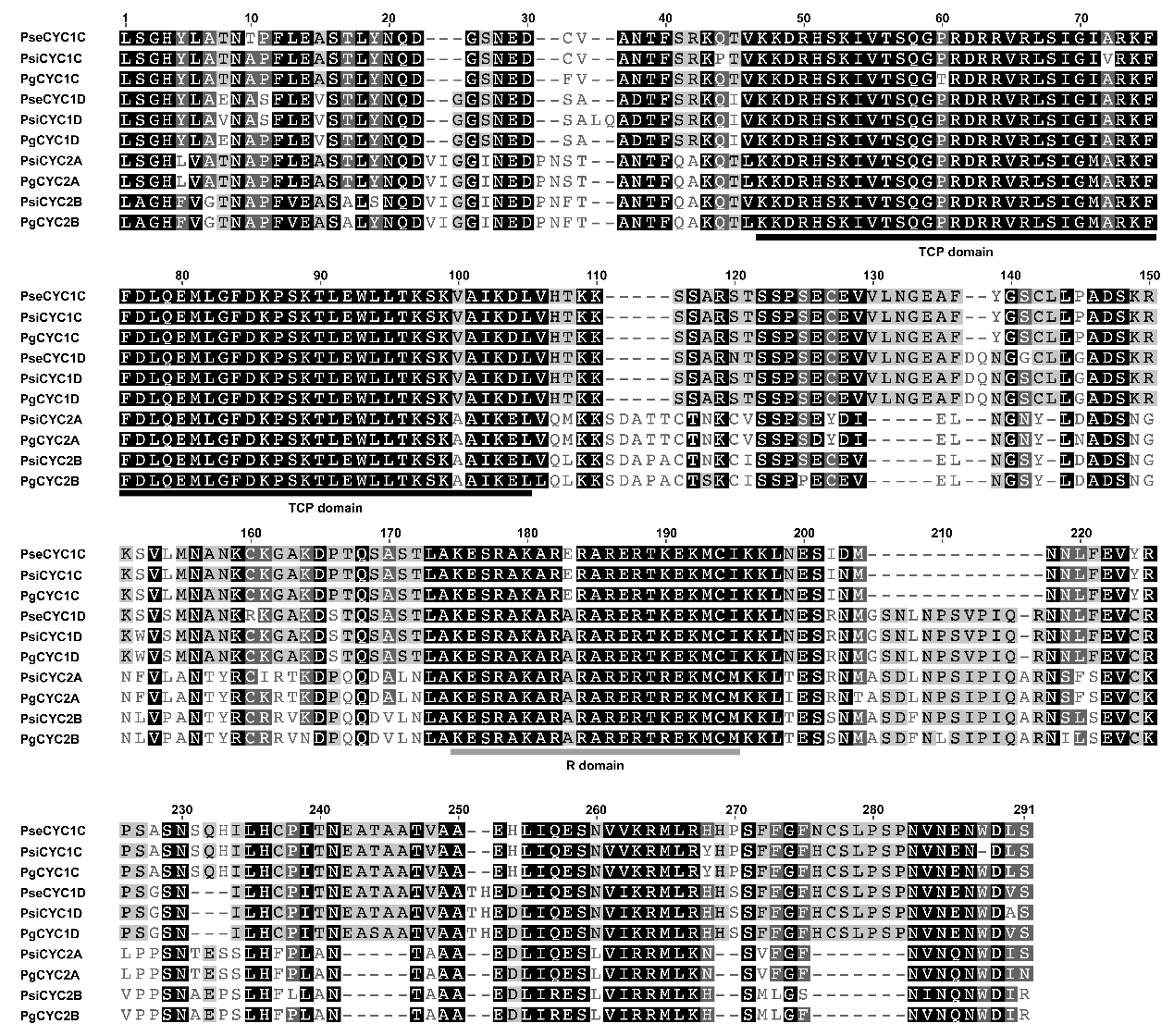


**Figure S4.** Sequence alignment of PseCYC1C/D and PgCYC1C/D with other related proteins. Sequences are from *Petrocosmea glabristoma* (Pg), *P*. *sericea* (Pse) and *P*. *sinensis* (Psi). The conserved TCP and R domains are marked by black and grey underlines, respectively. A hyphen represents a gap inserted to optimize alignment.


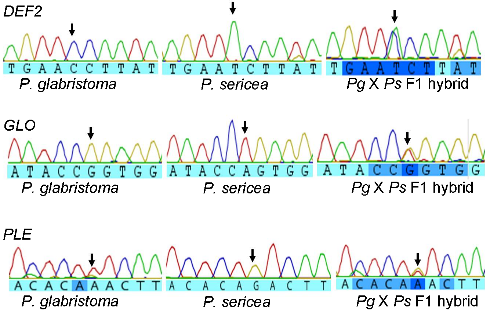


**Figure S5.** SNP identification of *DEF2*, *GLO* and *PLE* genes in *Petrocosmea glabristoma* and *P*. *sericea*. The arrow heads in parental species indicate the SNP sites between the two parents, while the arrow heads in the F_1_ hybrids indicate the heterozygous sites from the two alleles. *Pg*, *P*. *glabristoma*; *Ps*, *P*. *sericea*.


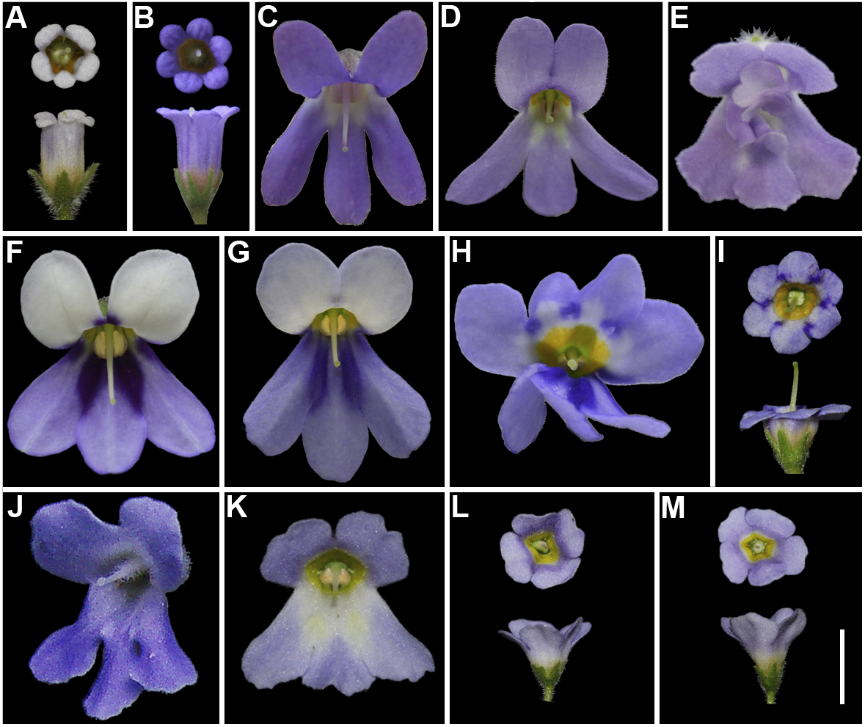


**Figure S6.** Morphology of wild-type and mutant flowers in the hybrids of *Petrocosmea glabristoma* crossed with other *Petrocosmea* species. (A, B) dorsalized flowers with pentamerous (A) and hexamerous (B) floral organs in face and lateral view; (C) flower of *P*. *nervosa;* (D, E) flower variation of F_1_ hybrids of *P*. *nervosa* × *P*. *glabristoma* with wild-type flower (D) and petaliod-stamen flower (E). (F) flower of *P*. *oblate*; (G-I) flower variation of F_1_ hybrid of *P*. *oblate* × *P*. *glabristoma*. (G) wild-type flower of F_1_ hybrid, (H) flower with four dorsal petals; (I) dorsalized hexamerous flower in face view and lateral view; (J) flower of *P*. *qinlingensis*; (K-M) flower variation of F_1_ hybrids of *P*. *qinlingensis* × *P*. *glabristoma*. (K) wild-type flower of F_1_ hybrid; (L, M) dorsalized flower with tetramerous (L) and pentamerous (M) floral organs in face and lateral view. Bar, 0.5cm.

**Table S1** Primers used for gene isolation in this study

| **Primer name** | **Sequence (from 5′ to 3′)** |
| --- | --- |
| GSQU-F | GTACAACTGAAGAGGATAGAGAAC |
| GSQU-R | GCTCTTGTATTGCCTTCTCC |
| GLIP-F | GTCACGTTTTACCGCCGAACTGG |
| GLIP-R | GTGGGAATCCTGATGATGCTGC |
| GPLE-F | CCTTCTGCAAACGTAGAAATGG |
| GPLE-R | GCATTGTGCAGCTCAAGCTCCC |
| GDEF-F | GGCAGGTCACCTACTCCAAGAG |
| GDEF-R | CTAACCTTTTTCTTGCTGGTGTC |
| GDEF-F2 | ATGGCTCGAGGAAAGATCCAGATCAAG |
| GDEF-R2 | CTACTCAAGCAAAGCAAAAGTGGTGAGATC |
| GGLO-F | GAATTGAGAACTCAAGCAACAGGC |
| GGLO-R | GAAGATTAGGCTGCATTGGCTG |
| PCYC1C-F | CTTTCCGGTCATTATTTAGCCAC |
| PCYC1C-R | ATGGGACAATGAAGAATATGCTG |
| PCYC1D-F | CTTTCCGGCCATTATTTAGCTGA |
| PCYC1D-R | GCAAACTTCAAACAAATTGTTCCTCTG |

**Table S2** Primers used for Real-time PCR in this study

| **Primer name** | **Sequence (from 5′ to 3′)** |
| --- | --- |
| PSQU-RT-F | GGTGACGTTCTCAAAGAGGAG |
| PSQU-RT-R | GCAAGAATCGGTAGCATACTC |
| PLIP-RT-F | GTCACGTTTTACCGCCGAACTG |
| PLIP-RT-R | CTCCTCGGAACTTAATGGCTGC |
| PDEF1-RT-F | CAGTACTCAAAAGCTTCACGAG |
| PDEF1-RT-R | CATTTTCTCATATTGAGAGCTCC |
| PDEF2-RT-F | GCTCATGAGCTTACTGTTCTC |
| PDEF2-RT-R | GTACTGATCAAACAACTGCTTC |
| PGLO-RT-F | GCTAAGGAGATCAGTGTTCTCTG |
| PGLO-RT-R | GCTCCAAAATGTCTGATAACG |
| PPLE-RT-F | GTTGTCTGTTTTGTGTGATGC |
| PPLE-RT-R | GAGCTTGCTTTCTTGTACCTGTC |
| PCYC1C-RT-F | CTTTCCGGTCATTATTTAGCCAC |
| PCYC1C-RT-R | CTGTGCCTATCTTTTTTCACGG |
| PCYC1D-RT-F | GCTTTCCGGCCATTATTTAGCTG |
| PCYC1D-RT-R | TTGAAAATGTGTCGGCCAGGG |
| PACT-RT-F | CTCGTGAATACCAGCAGATTCC |
| PACT-RT-R | CCAAGAGCAGCTCTTCCATAGAG |

**Table S3** Primers used for allele-specific expression in this study

| **Primer name** | **Sequence (from 5′ to 3′)** |
| --- | --- |
| PgDEF2-RT-F | CTGGACTACCATCAAATGGTGAAC |
| PsDEF2-RT-F | GGACTGCCATCAAATGGTGAAT |
| PhDEF2-RT-R | TTGCCAGTCTCGATCTGGTTG |
| PgGLO-RT-F | CATCTGGATCCGATGGATACCG |
| PsGLO-RT-F | CATCTGGATCCGATGGATACCA |
| PhGLO-RT-R | GGCAGGCTTCATAATTTGCAACC |
| PhPLE-RT-F | CCCTCCAATACTGTGTCAACATC |
| PgPLE-RT-R | CCAAGAATTTGCCTGTTTGAAGTT |
| PsPLE-RT-R | CAAGAATTTGCCTGTTTGAAGTC |
